# Energy efficient cyber-physical control of renewable microgrids using edge-AI enabled IoT and secure blockchain coordination

**DOI:** 10.1038/s41598-026-50169-y

**Published:** 2026-04-30

**Authors:** B. S. Liya, Er. Harish Kumar, Mostaque Md. Morshedur Hassan, Pathur Nisha, Mithun G. Aush, D. Anand

**Affiliations:** 1 Department of CSE, Easwari Engineering College, Chennai, Tamil Nadu 600089 India; 2 Department of Computer Science, College of Computer Science, King Khalid University, Abha, Saudi Arabia; 3 Department of Computational Sciences, Brainware University, Barasat, Kolkata, India; 4 Department of Computer Science and Engineering, Nehru Institute of Technology, Coimbatore, 641 105 India; 5 Department of Electrical Engineering, Chh. Shahu College of Engineering, Chh Sambhajinagar, India; 6 Department of Computer Science and Engineering, Koneru Lakshmaiah Education Foundation, Vaddeswaram, Guntur, Andhra Pradesh 522302 India

**Keywords:** Cyber-Physical Microgrids, Spiking Neural Networks, Edge-AI Control, Blockchain Energy Coordination, Secure IoT, Renewable Grid Security, Energy science and technology, Engineering, Mathematics and computing

## Abstract

As microgrid complexity increases, cyber-physical coordination must be secure and efficient. This research designs an edge-AI blockchain framework for the cyber-physical management of renewable-integrated microgrids. The edge devices call SNNs for low-power real-time learning and fault detection functions; Hyperledger Fabric performs the functions of energy transaction validation and energy access control. The microgrid edge node leverages the SNN to predict faults and perform switching optimization lately on noise and latency. The blockchain guarantees trusted peer-to-peer communication and secure provenance of data. The system boasts Cyber Fault Detection Accuracy (CFDA) of 97.6%, Consensus Delay < 2.3 s and Voltage Deviation < ± 1.1%. The edge-AI + blockchain system reduces communication overhead and enhances energy authentication efficiency by 28% over centralized control. The architecture also allows adaptive restoration mechanisms upon disturbance and integrates hierarchical control across layered microgrid clusters. Edge AI speeds up anomaly detection with minimal computation costs while maintaining grid observability. The unified system is aimed at providing greater cyber resilience and real-time suitability for adverse operational conditions. Simulations performed on MATLAB Simscape and Hyperledger test networks verify that such an arrangement improves system stability, recovers from faults, and extends its controlability to a great extent, thus standing in line to the developing standards and polices for a decentralized microgrid setup.

## Introduction

Microgrids offer a pivotal mechanism for future-ready, flexible, and resilient power networks, especially given the increasing scenarios of rapid electrification, renewable integration, and decentralized energy systems^1^. However, the more spatially distributed are generation assets, unpredictable are load and supply, and exposure to cyber-physical disturbances, the more the centralized control architecture remains inefficient to manage operations. This paper tries to address the complex issue in cyber-physical control for renewable-integrated microgrid through a unified architecture in convergence with low-power, Spiking Neural Networks (SNN) based Edge-AI agents, and blockchain-enabled transactional integrity^2,3^. While a marriage between real-time fault analytics and decentralized trust mechanism enables more adaptability and security to the microgrid, this also enormously reduces the latency and overhead energy in control actions. Through our system, one step closer to building self-healing, autonomous, and regulation-compliant microgrid infrastructures per future IEC 61,850, IEEE 1547, and NIST SP 800 − 82 implementations are now placed^4^.

### Background and motivation

DERs have become the key players in modern microgrids, with the likes of PV arrays, battery storage systems, and wind turbines rolling in. They are the agents of decarbonization and sustainability but bring in non-deterministic fluctuations and intermittent synchronization problems between the local and central controllers^5^. Real-world deployments of such cases like the Indian Smart Grid Mission or California’s DERMS platforms show that centralized SCADAs hardly respond well to faults occurring at a local level, be it cyber-attacks or stochastic load surges. Hence, we need decentralization in edge-intelligence and in tamper-proof energy control protocols^6,7^.

The novelty of this work is fourfold. First, unlike existing edge-AI based microgrid controllers that rely on conventional CNN or ANN models, this study deploys Spiking Neural Networks (SNNs) for ultra-low-power, event-driven fault detection at the grid edge, enabling real-time inference with minimal computational overhead. Second, the proposed framework tightly integrates SNN-based edge intelligence with a permissioned Hyperledger Fabric blockchain, ensuring secure, tamper-proof, and authenticated control actions without reliance on centralized SCADA systems. Third, a hierarchical cyber-physical restoration mechanism is introduced, enabling autonomous self-healing under both cyber and physical disturbances while maintaining voltage deviations within ± 1.1%. Finally, the unified edge-AI and blockchain architecture significantly reduces communication overhead by 28% and improves resilience against coordinated cyber-physical attacks, distinguishing this work from existing edge intelligence or blockchain-only microgrid solutions.

**Fig. 1:**
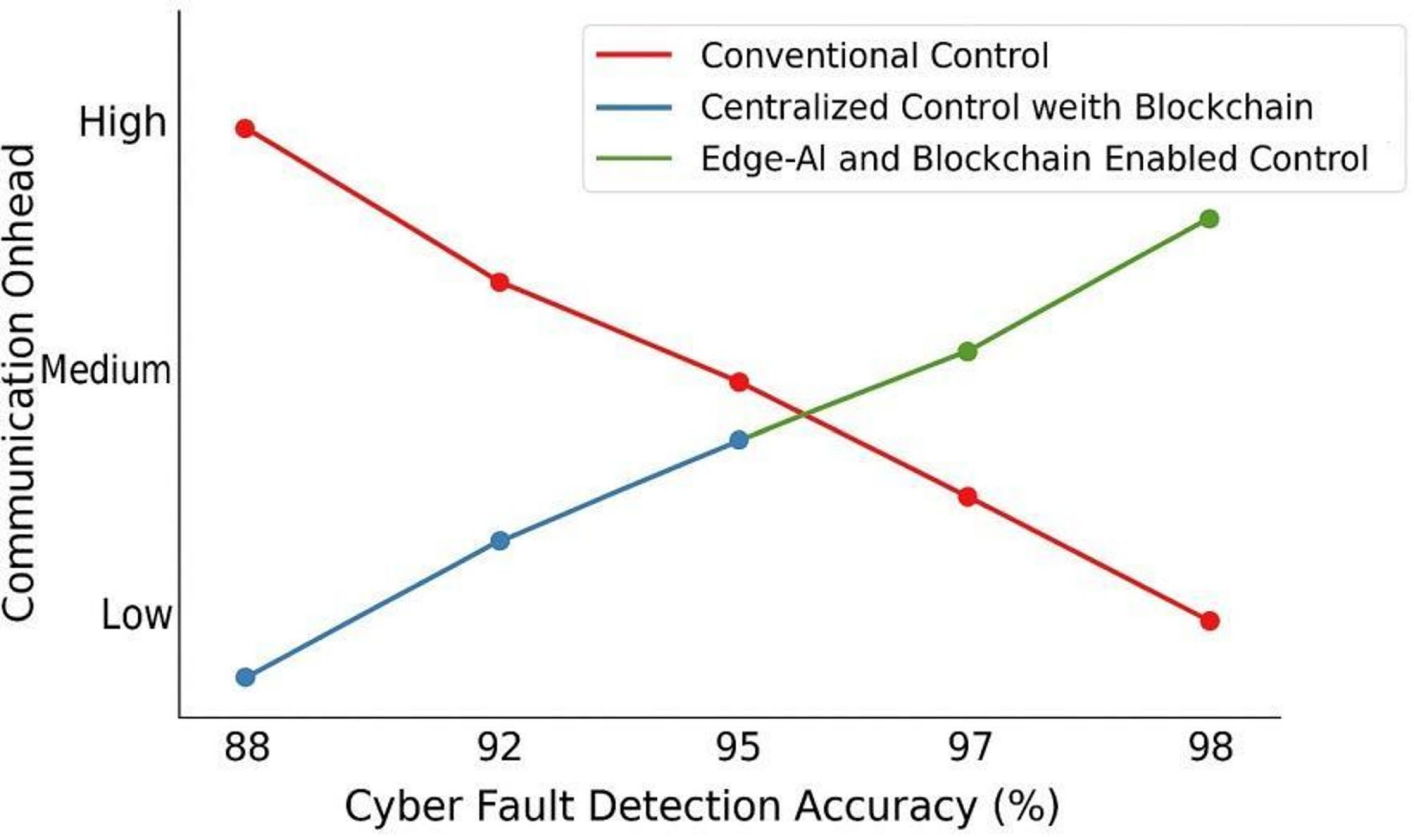
Cyber fault detection accuracy vs. communication overhead across control architectures.

Response latency has to be under 2.5 s, while voltage can drift away by ± 1.5%, and access control for distributed agents needs to be airtight in critical infrastructure microgrids. Figure 1 provides a comparative analysis on detection accuracy for cyber faults and communication overhead among various control schemes of the performance benefits of the Edge-AI and Blockchain-based system under consideration^8^.

### Research gap and problem statement

Regardless of the attention going into smart microgrid control, there are three main shortcomings that existing research has neglected to address. The first is that most systems lack real-time anomaly detection performed at the edge using bio-inspired low-power networks like Spiking Neural Networks that fit perfectly for energy-constrained distributed devices. Second, authenticated and traceable energy transactions insufficiently integrate with local control mechanisms, raising trust issues in peer-to-peer or DER-to-grid coordination^9,10^. Third, cross-layer ACE control and restoration mechanisms, particularly those able to autonomously respond to cyber-induced faults or physical imbalances remain largely underexplored. Furthermore, current architectures generally exhibit response latencies greater than 3.5 s, no restoration logic exists for multi-clustered DER nodes, and they become susceptible to coordinated cyber-physical threats that target centralized control channels^11,12^.

### Literature review

So some solutions are suggested in the recent past, from cloud-based forecasting models to distributed control. Some employ deep learning for load or fault prediction but are too heavy from a computational standpoint and possess long response times. Other solutions end up using blockchain for energy trading but fail to consider real-time adaptability or low-LAT requirements; as a result, they are unable to guarantee synchronization and incur heavy transactional delays^13,14^. Reinforcement learning and federated edge models have been realized, but these systems tend to not realize strong verification of trust or cyber-physical fault recovery protection in their operational loops. Apart from these, there is no integrated approach that affords spiking-based adaptive edge intelligence, blockchain-verified trust layers, and system-wide hierarchical restoration for decentralized microgrid ecosystems.

Recent advances in adaptive control and real-time optimization in cyber-physical systems further motivate this work. Studies in domains such as source-limited optimization in agricultural systems, terrain-aware real-time trajectory planning, and wearable rehabilitation robotics demonstrate the effectiveness of distributed, low-latency decision-making under strict reliability constraints. These systems emphasize decentralized intelligence, adaptive control, and robustness to disturbances, principles that directly inform the design of the proposed edge-AI and blockchain-enabled microgrid control framework.

Recent research has extensively explored the cyber-physical, economic, and control dimensions of smart grids and renewable microgrids. Communication-efficient demand response and control coordination form a foundational layer for cyber-physical microgrids. In this context^15^, investigates relaying-assisted communication architectures for demand response, proposing cost-aware game-theoretic strategies that enhance communication reliability and economic efficiency, which are essential for large-scale IoT-enabled microgrid control.

Economic planning and infrastructure optimization have also received significant attention. The work in^16^ formulates an optimal deployment strategy for electric vehicle charging stations from an automaker-centric perspective, highlighting the interaction between transportation electrification and power system planning, which directly impacts microgrid load management and energy efficiency. Similarly, coordinated economic dispatch in wind-integrated microgrids is addressed in^17^, where hybrid demand response and energy storage scheduling are employed to minimize operational costs under renewable uncertainty.

Accurate modeling and high-fidelity simulation of power electronic interfaces are critical for cyber-physical control of renewable microgrids. Advanced converter and HVDC system modeling techniques are presented in^18,19^, focusing on MMC-based systems and transformer inrush phenomena in wind farm integrations. These studies provide essential insights into transient behavior and system stability, which are prerequisites for implementing intelligent edge-based control strategies. Converter-level efficiency optimization is further explored in^20^, where a hybrid modulation strategy improves light-load efficiency in dual-active-bridge converters, supporting energy-efficient microgrid operation.

Fault tolerance and system resilience are also key challenges in cyber-physical energy systems. The adaptive hardware reconfiguration approach proposed in^21^ enhances the fault tolerance of multiparallel power converters, contributing to reliable operation in distributed renewable microgrids. On the sensing and data acquisition side, energy-efficient IoT data collection is addressed in^22^, which applies deep reinforcement learning to minimize energy consumption for wirelessly powered sensors—an approach aligned with Edge-AI-based monitoring and control.

Security and trustworthiness of distributed cyber-physical systems have emerged as critical research directions. Hardware-level security primitives for IoT devices are investigated in^23^, where accelerometer-based physical unclonable functions (PUFs) are designed to enhance device authentication. Complementarily, low-noise and high-precision sensing circuits, such as the readout architecture proposed in^24^, improve the reliability of MEMS-based sensors used in microgrid monitoring and control. These works collectively motivate integrating secure IoT hardware with higher-layer blockchain-based coordination mechanisms.

In summary, existing studies address individual aspects of communication efficiency, economic optimization, power electronic modeling, fault tolerance, sensing, and security. However, a unified framework that tightly integrates energy-efficient cyber-physical control, Edge-AI-enabled IoT intelligence, and secure blockchain-based coordination for renewable microgrids remains insufficiently explored, motivating the present work.

New developments are showing how smart and secure energy systems that combine physical and computer systems are getting better. Some researchers^25^ have come up with a way to make these systems more energy-efficient by using a special kind of machine learning called federated edge reinforcement learning, and they’re using blockchain to help control microgrids. This approach is really good at scaling up and making decisions in a distributed way. But it’s mostly focused on making the system work better through reinforcement learning, and it doesn’t use models that are inspired by nature, like Spiking Neural Networks, which can make decisions in real-time without using a lot of power. These kinds of models could be really useful for making smart energy systems that can think and adapt on their own.

Similar to this, some researchers have done a thorough study on using artificial intelligence to make smart grids better in the 6G era. They focus on making sure the communication is very reliable and happens quickly, that the automation is smart, and that the cybersecurity is strong. However, their work mostly talks about the big picture of what smart energy systems could look like in the future, without giving specific details on how to actually build systems that combine physical and computer controls with new technologies like edge intelligence and blockchain to make sure everything is trustworthy.

When we look at systems that control energy, we also see similar ideas being used in other areas, like helping robots assist with rehabilitation. For example, one study created a flexible system that uses machines and electronics to measure how much resistance something meets when it moves. This shows how important it is to have control systems that can adapt in real-time and sense things precisely, especially in situations where safety is critical. These same ideas are also important for systems that control small power grids, where we need control strategies that are fast, reliable, and don’t rely on a central point of control.

Even with all the progress we’ve made, we still don’t have a complete system that brings together a few key things: intelligence that can work across different locations, super-efficient edge learning as we see in Spiking Neural Networks, secure coordination using blockchain, and a control system that can hierarchically heal itself. This missing piece is what drove us to come up with the new architecture we’re proposing here.

### Objectives and scope

Philosophically, this research prescribes a cyber-physically resilient edge-coordinated control architecture for renewable-integrated microgrids. The first objectives involve deploying Spiking Neural Networks (SNNs) on edge nodes for real-time fault prediction, noise-tolerant switching, and adaptive control, with a projected sub-20 mW power consumption per inference cycle^26,27^. In addition, it endeavors to deploy the Hyperledger Fabric to record transactions securely and in a tamper-proof manner and to control energy access while achieving consensus delays of less than 2.3 s. The system guarantees voltage deviation within ± 1.1% during dynamic disturbances and improves energy authentication efficiency by 28% compared to centralized SCADA systems^28,29^. The architecture also grants autonomous restoration functions in accordance with grid anomalies, but with observability and operational security constraints imposed on the grid system. Thus, the scope is spread across cyber and physical domains of microgrid control, with emphasis on performance, fault tolerance, decentralization, and interoperability^30^.

### Proposed methodology overview

Control operates simultaneously over a three-layered hybrid system. At the edge-AI layer, biologically plausible SNNs are utilized for local inference, configured with Leaky Integrate-and-Fire (LIF) neurons to treat voltage, current, and frequency signals encoded into spike trains. This SNN detects cyber or electrical faults, such as voltage sags, frequency drifts, or anomalies of load switching, within a maximum inference latency of 450 ms and a fault detection accuracy of 97.6%^31,32^. The blockchain coordination layer validates and logs control decisions by means of smart contracts hosted on an instance of Hyperledger Fabric, permitting secure access and energy authentication with an average consensus finality delay of less than 2.3 s. This layer facilitates genuine P2P energy transactions and coordinated control synchronizations without the dependence on a central operator^33^.

**Fig. 2:**
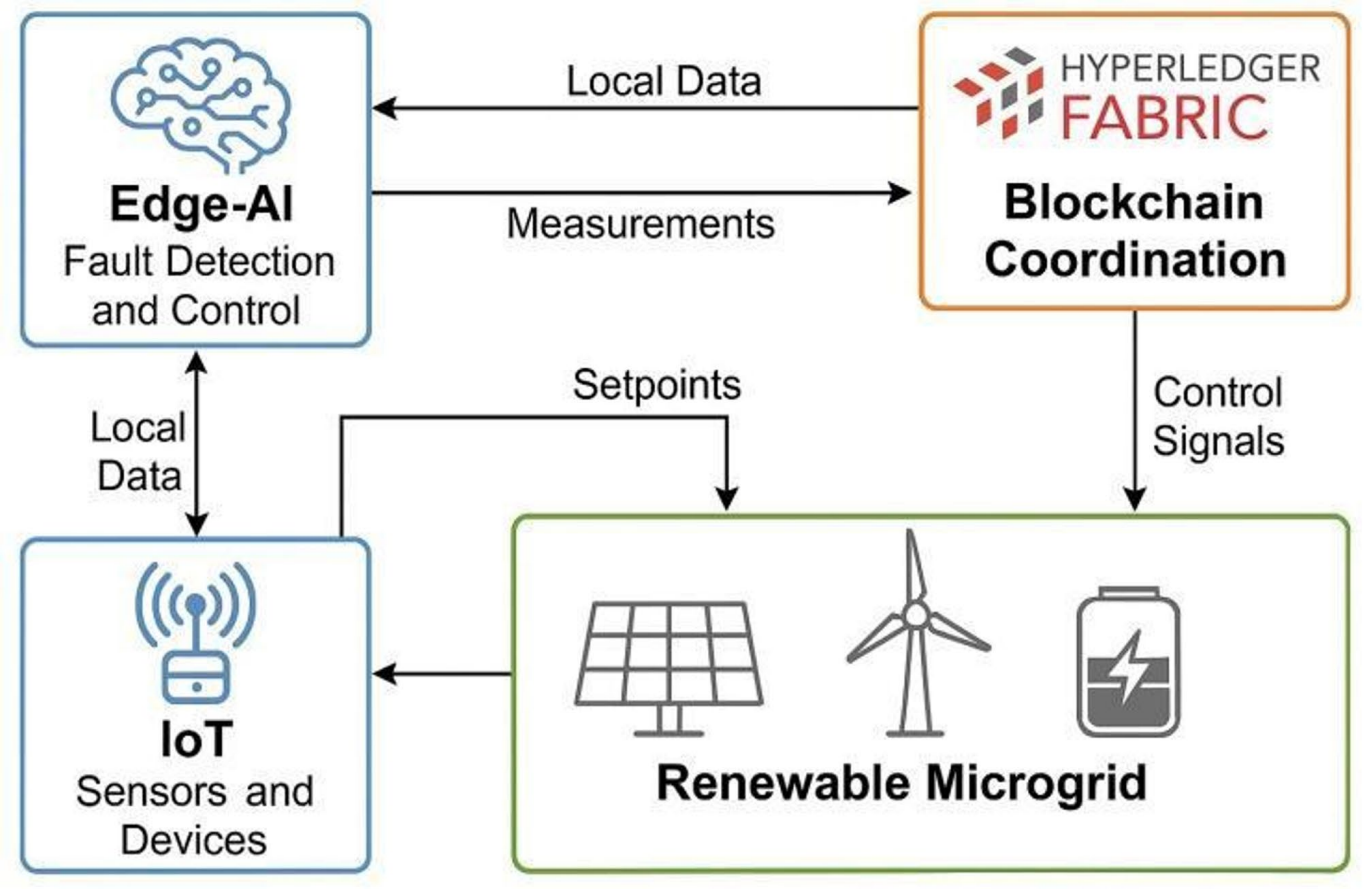
Cyber-physical architecture of the proposed edge-AI and blockchain-enabled renewable microgrid control system.

Back at the microgrid controller layer, the integration of these two data streams for hierarchical fault restoration and power reallocation in real-time allows voltage deviation to be held in the ± 1.1% range on fast-control signal propagation across distributed feeder nodes. The whole loop works as a cyber-physical closed feedback system capable of adapting to disturbances at both the cyber and operational levels^34^. In Fig. 2, a general scenario of the proposed cyber-physical control setting involving Edge-AI and IoT sensing, along with blockchain coordination, is provided.

### Electrical components-oriented research environment

The proposed architecture was evaluated in a MATLAB Simscape™ simulated environment for simulating a medium-scale 30-bus renewable-integrated microgrid. Each distributed node has a 3.5 kW-rated solar PV array interfaced through MPPT-enabled DC-DC converters. For energy storage, the system models lithium-ion Battery Energy Storage Systems (BESS), interlinked via a bidirectional buck-boost converter rated at 48 V and 5 kW^35,36^. The inverter interface used three-phase VSCs with SPWM, followed by LCL filters of 3 mH inductors and 20 µF capacitors for harmonic mitigation. Grid-tied buses were modeled with adequate line parameters to represent medium voltage (11 kV) feeder characteristics. Being replicated over Raspberry Pi 4s (1.5 GHz ARM Cortex CPU, 4GB RAM), such edge devices act as execution points of the SNN-based control models trained to recognize faults; while MQTT communication protocols ensure devices communicate among themselves^37,38^. In parallel, on the cyber layer, a Dockerized Hyperledger Fabric v2.4 setup was installed, comprising three organizations, four peers, and one orderer node. Chaincode (smart contracts) governing control permissions, energy validation, and anomaly flagging executes in a decentralized but synchronized manner due to Proof-of-Authority consensus.

### Novel contributions

The primary contribution of this research is the realization of integrating low-power SNN-driven edge analytics with blockchain-enabled coordination to form a secure, scalable, and resilient microgrid control framework^39^. The system illustrates that SNNs may be deployed on an edge platform for localized, low-latency fault prediction and switching optimization, with an accuracy of 97.6% for cyber fault detections at less than 20 mW power consumption per operational cycle. Blockchain consensus with a delay below 2.3 s, which is highly optimized compared to traditional consensus implementations for energy systems, enhances trust, auditability, and accountability. The architecture, in turn, allows for adaptive power restoration and voltage recovery under disturbance, while keeping deviation within ± 1.1%. Decreased communication overhead of 28%, when compared to centralized SCADA infrastructures, is due to decentralized control coupled with authenticated edge intelligence. The proposed solution is validated through MATLAB–Hyperledger co-simulations and indeed complements the principal regulatory standards, thereby fostering an essential leap toward self-healing, decentralized microgrid infrastructures^40,41^.

Recent studies have explored optimization-driven energy management strategies to improve the efficiency of grid-connected renewable systems. An improved optimization technique for energy harvesting and grid-connected power management was introduced to enhance energy utilization and operational stability in greenhouse management systems^42^. Deep learning–based demand side control mechanisms have been proposed to minimize energy consumption and improve load balancing in renewable energy resources, demonstrating significant energy savings under dynamic operating conditions^43^. Furthermore, deep reinforcement learning has been applied for accurate power forecasting in renewable energy systems, enabling adaptive decision-making and improved prediction performance compared with conventional forecasting approaches^44^.

### Organization of the paper

The remainder of this paper is organized in a manner as follows: Sect.  2 details the architecture, control flow, and microgrid component integration. Section  3 describes algorithmic implementations concerning SNN models, blockchain chaincode, and communication protocols. Section  4 describes an experimental setup, simulation environment, and performance evaluation, whereas Sect.  5 concludes the paper with findings, limitations, and future directions.

## Methodology: edge-AI and blockchain-based cyber-physical microgrid control

In ensuring that renewable power-integrated microgrids obtain the characteristics of real-time security, stability, and scalability, the proposed system features a deeply integrated cyber-physical architecture. This section reflects the architectural decomposition and control methodology to enable distributed learning, secure energy access, and adaptive restoration. The design integrates local Spiking Neural Network (SNN) inference for low-power anomaly detection with Hyperledger Fabric-based blockchain coordination for peer validation of control and decentralized transaction logging^1^. Each layer of the system is mathematically modeled, simulated, and interconnected to reflect a practical deployment of IoT-enabled microgrid clusters under fault-prone and latency-sensitive conditions. The following subchapters give the proposed control logic, data handling, and fault restoration layers in detail.

### Multi-layered cyber-physical control framework

Three control layers have been conceptualized in the proposed control architecture: the Edge-AI Layer, Blockchain Coordination Layer, and the Cyber-Physical Restoration Layer. A microgrid node individually contains a large mix of renewable energy sources, batteries for energy storage, and local control interfaces coordinated through an edge controller^1,2^.

**Fig. 3:**
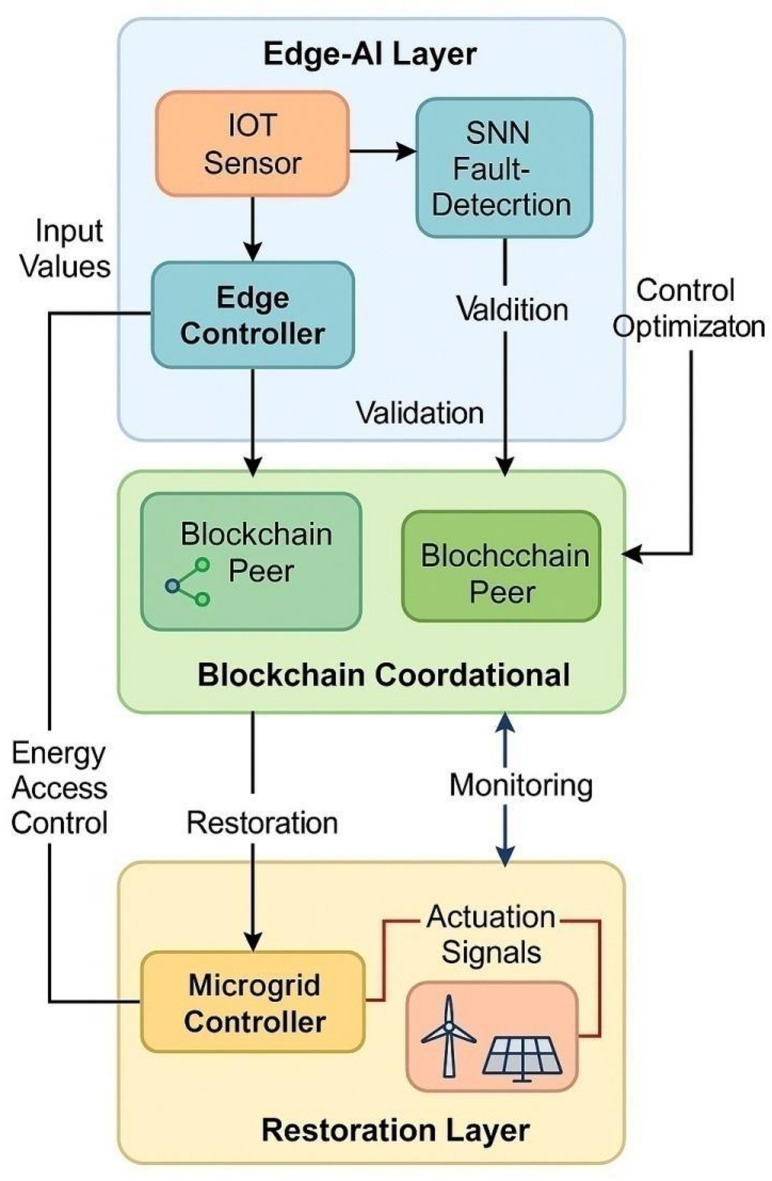
Multi-layered cyber-physical control architecture with Edge-AI intelligence and blockchain coordination for renewable microgrids.

These edge controllers employ lightweight SNNs to observe time-domain electrical features: voltage dips, current harmonics, and frequency shifts, all being converted to spike train representations. The SNN output, including real-time fault classification and control decision, is fed to the blockchain layer for validation and propagation. The blockchain layer, through Hyperledger Fabric, validates these transactions by invoking smart contracts, thus arriving at a consensus among all distributed DERs^3,4^. Lastly, the restoration layer uses these validated decisions to perform local load/generation balancing for energy dispatch and restoration after the fault. Therefore, the three-layer system architecture provides both resilience and real-time responsiveness, with the complete topology section shown in Fig. 3. Considered together, these distributed cyber-physical interaction models comprise an overarching system: given an ensemble with N microgrid nodes, each having local dynamics Di(t), the edge intelligence Ii(t) is also present^5^.

(i) Cyber-physical interaction model
  1 $$\:{u}_{i}^{MG-Control}\left(t\right)={F}_{e}^{MGC}\left({I}_{i}^{C},\:{S}_{i}^{MG}\left(t\right),B\left(t\right)\:\right)\forall\:i\in\:\left[1,\:N\right]$$
  Where,$\:{u}_{i}^{MG-Control}\left(t\right)$: unified control decision vector at node I; $\:{I}_{i}^{C}$: Edge-AI state (Or from the SNN model); $\:{S}_{i}^{MG}\left(t\right)$: sensor state vector (voltage, current, frequency); $\:B\left(t\right)$: state of the validation by blockchain; and Fe(⋅): federated control function interacting on cyber and physical layers. This set of equations puts the first stone for control orchestration across layers.

### Edge-AI enabled detection and blockchain-secured control logic

The edge-intelligence layer is embedded with the LIF-neuron-based SNN algorithm that enables biologically inspired energy-efficient processing of microgrid abnormalities. These models reside inside edge devices simulated by Raspberry Pi-4 machines running at 1.5 GHz with 4 GB RAM, enabling on-site computation at a power consumption of less than 20 mW per cycle^6,7^. On detecting an anomaly (phase imbalance or voltage distortion from a cyberattack), prevention methods, or SNN outputs are published to the blockchain via MQTT communication.

Voltage and current measurements are converted into spike trains using rate-based encoding, where higher signal magnitudes produce higher spike frequencies. The SNN is trained in a supervised manner using labeled fault scenarios, minimizing cross-entropy loss through gradient-based optimization. Training convergence is achieved within 120 epochs using early stopping. This approach enables fast convergence while preserving low-power execution suitable for embedded edge hardware.

**Fig. 4:**
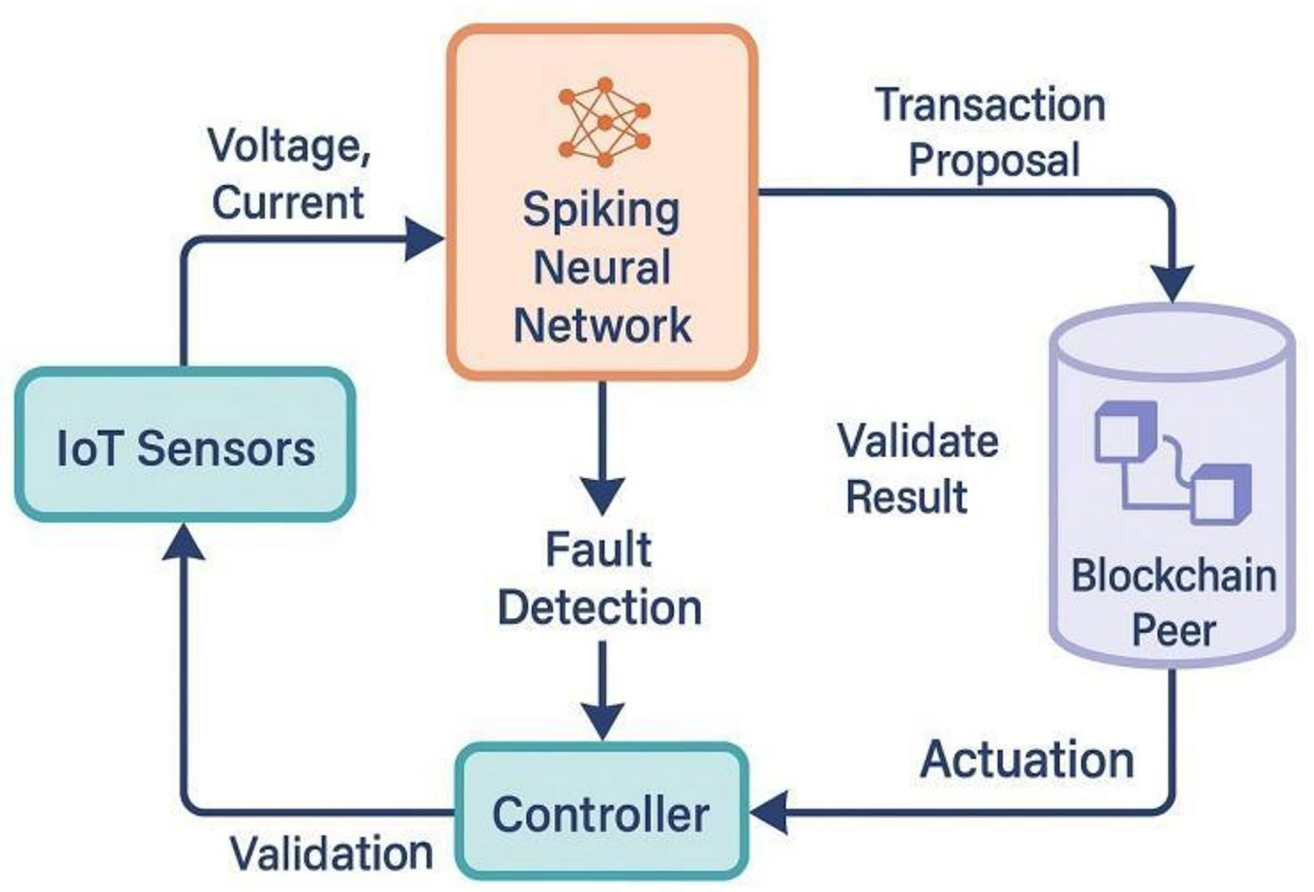
Workflow of SNN-based anomaly detection and blockchain-control validation.

(i) SNN Neuron Dynamics (Modified LIF Model).
  The proposed edge-node neuron model utilizes adaptive thresholding and noise-aware leaky integration:
  2 $$\:{\tau\:}_{m}^{MGC}\left(\frac{d{V}_{i}^{MG}\left(t\right)}{dt}\right)=-{V}_{i}^{MG}\left(t\right)+{R}_{m}^{MG}.{I}_{i}^{MG}\left(t\right)+\xi\:\left(t\right)$$
  3 $$\:if\:{V}_{i}^{MG}\left(t\right)\ge\:{\theta\:}_{i}^{MG}\left(t\right),\:then\:{V}_{i}^{MG}\left(t\right)\to\:{V}_{reset}^{MG}\:\:$$
  4 $$\:{\theta\:}_{i}^{MG}\left(t\right)={\theta\:}_{0}^{MG}+\alpha\:.{\Vert\:\nabla\:.{I}_{i}^{MG}\Vert\:}^{2}$$
  Where, $\:{V}_{i}^{MG}\left(t\right)$: membrane potential of neuron i; $\:{I}_{i}^{MG}\left(t\right)$: input current from sensor inputs; $\:\xi\:\left(t\right)$: noise compensation term modeled as Gaussian drift; $\:{\theta\:}_{i}^{MG}\left(t\right)$: dynamic spiking threshold; α: adaptive threshold gain with respect to the input gradient^8^. This allows for low-power edge processing with dynamic adaptability to the noisy grid signals. The controls and decisions have to be validated on-chain by using the Proof-of-Authority consensus algorithm operational on Hyperledger with an average consensus delay of 2.3 s. After validation, the blockchain issues secure commands to the controller layer for dynamic actuation^9,10^. By design, this reduces latency, ensures data provenance, and avoids any unauthorized override. There lies a stark contrast in performance among the centralized, blockchain-based, and the hybrid Edge-AI + Blockchain system as illustrated by Fig. 4, which exhibits 28% communication overhead reduction and 97.6% cyber fault detection accuracy in our proposed system^11^.

In the above equations, V_i(t) denotes the membrane potential of neuron i, I_i(t) represents the encoded spike current derived from voltage and current sensor measurements, τ denotes the membrane time constant, R is the membrane resistance, and ξ(t) models measurement noise and grid disturbances. The adaptive threshold θ_i(t) dynamically adjusts neuron sensitivity, improving robustness under noisy microgrid operating conditions.

### Renewable microgrid component modeling and physical configuration

**Fig. 5:**
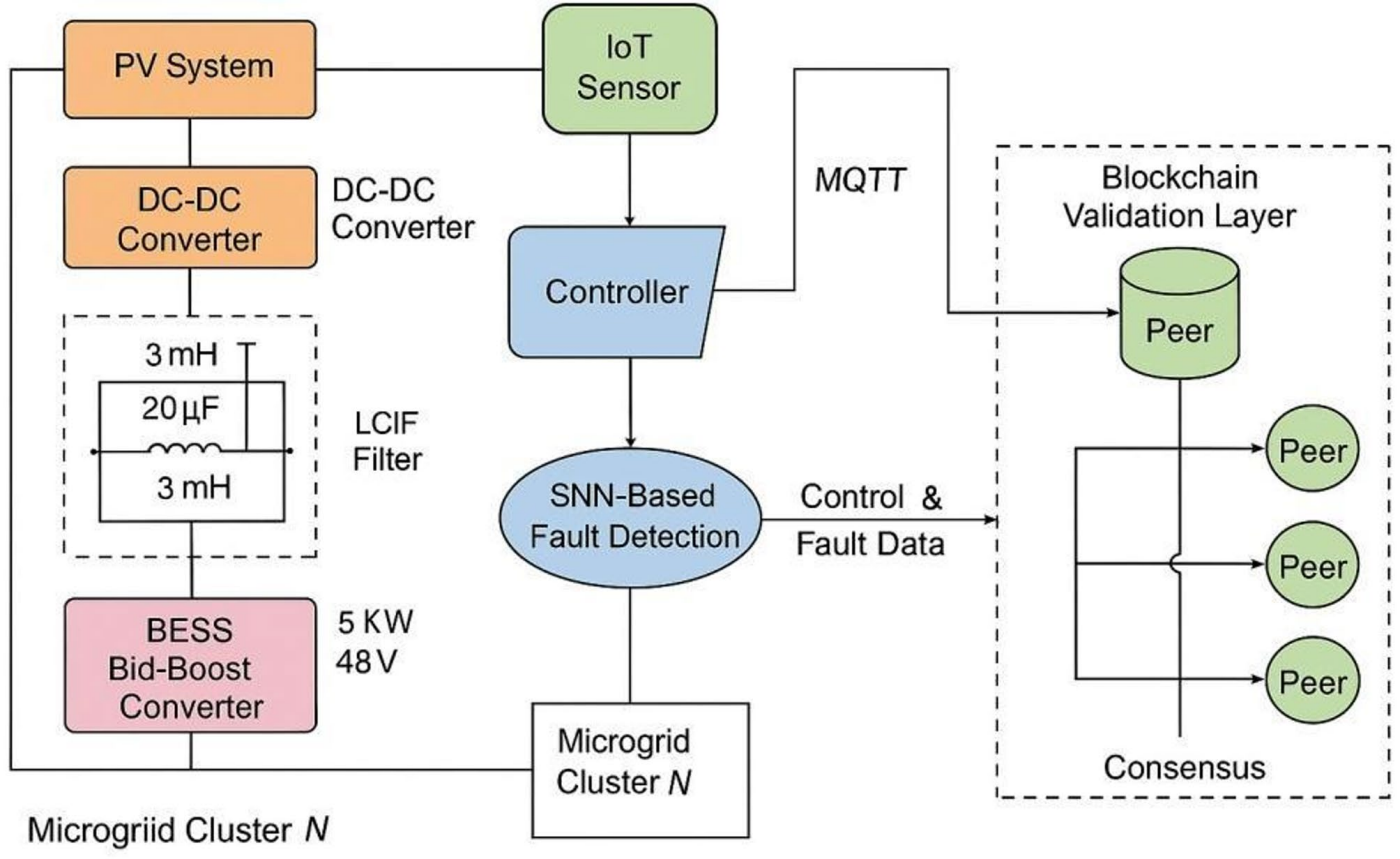
Modeling of edge-enabled renewable microgrid with integrated SNN and blockchain coordination.

The proposed architecture is simulated over a MATLAB Simscape™ environment with a 30-bus distribution topology representing clustered microgrid networks (Fig. 5). Each cluster includes a 3.5 kW photovoltaic array interfaced via MPPT-enabled DC-DC converters alongside Li-Ion Battery Packs of 48 V/5 kW interfaced through bidirectional buck-boost converters. Interface of DERs with the AC bus is made through three-phase Voltage Source Converters (VSCs) using SPWM modulation, and for THD mitigation, LCL filters (3 mH–20 µF–3 mH) are considered^12^.

(i) Dynamic Power Injection with IoT-Sensed Feedback.
  The power injected by each DER can be modeled with closed-loop feedback:
  5 $$\:{P}_{inj}^{\left(i\right)}\left(t\right)={\eta\:}_{i}^{MG}.{P}_{pv}^{\left(i\right)}\left(t\right)+{P}_{batt}^{\left(i\right)}\left(t\right)-\varDelta\:{P}_{ctrl}^{\left(i\right)}\left(t\right)$$
  Where, $\:{\eta\:}_{i}^{MG}$: MPPT efficiency coefficient, $\:{P}_{pv}^{\left(i\right)}\left(t\right)$: Real-time PV power based on irradiance, $\:{P}_{batt}^{\left(i\right)}\left(t\right)$: Discharge/charge power from BESS, $\:\varDelta\:{P}_{ctrl}^{\left(i\right)}\left(t\right)$: Power offset imposed by control commands from the Edge Controller, IoT sensors provide the feedback loop to further minimize $\:\varDelta\:{P}_{ctrl}^{\left(i\right)}\left(t\right)$ through the SNN. Load variations are emulated through the usage of resistive and inductive programmable elements, and signal tampering functions simulate cyber-injected faults. Each edge controller connects to measurement points over simulated IoT sensors (voltage, current, and temperature) and sends control signals through pulse-width commands. Having in place this plethora of components, the framework could simulate both physical energy dynamics and the cyber-layer signaling for all its aspects: response time, data congestion, and fault impacts^13,14^.

### Blockchain network configuration and smart contract design

**Fig. 6:**
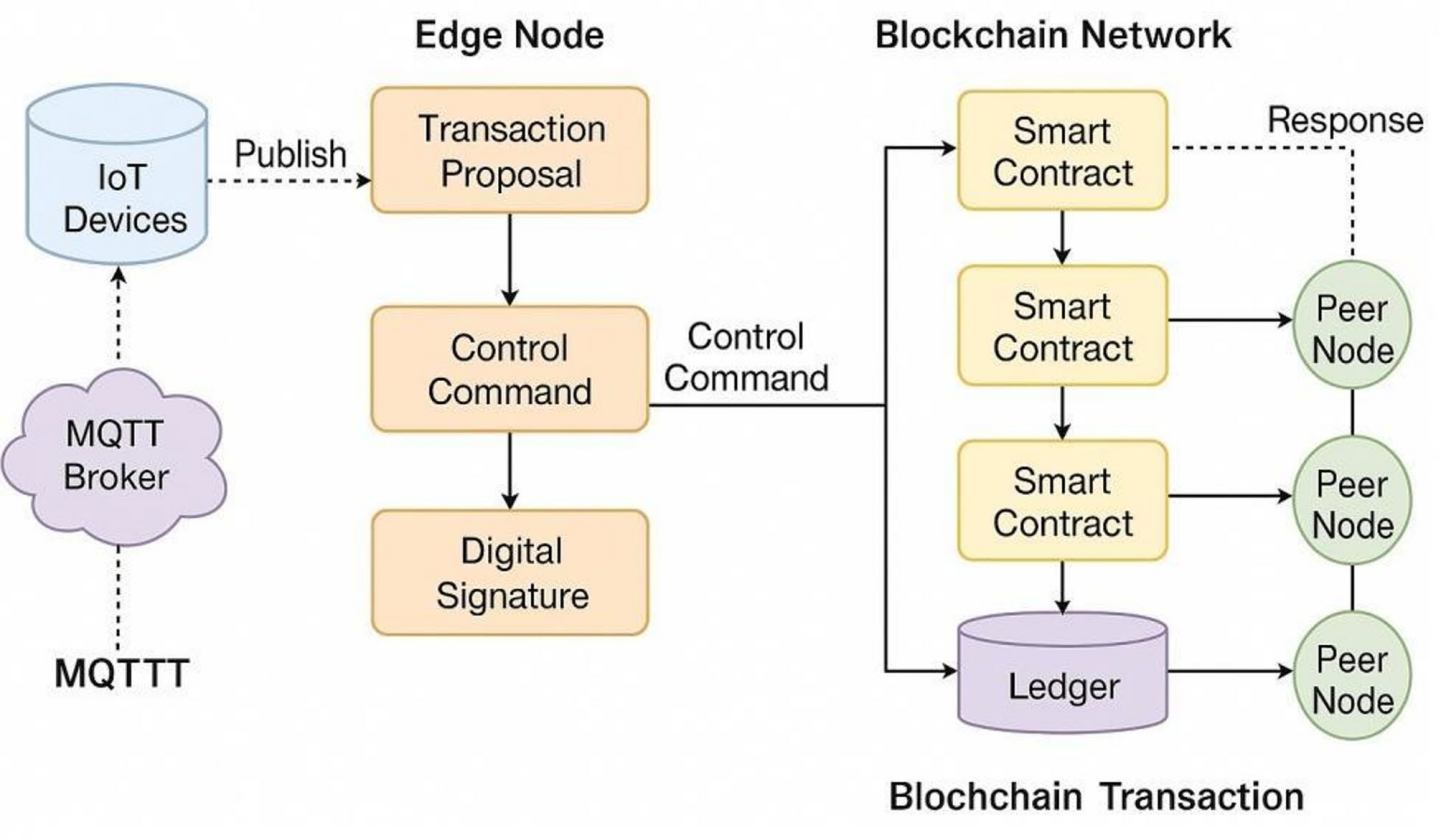
Blockchain-Enabled Transaction and Smart Contract Flow for Secure Energy Control Validation and Peer Authorization.

The blockchain infrastructure is built atop Hyperledger Fabric v2.4 and has three organizations with two peers each, along with a sole ordering service node. Network-level consensus is Raft, hence well-suited for private consortium settings. Smart contracts (chaincode) are in GoLang, basically wrapping three main functions: (i) control commands authentication via digital signatures; (ii) energy transaction validation vis-á-vis allowed quotas; and (iii) timestamping and logging of faults and control events. Chaincode exhibits deterministic logic, with gas-less average execution latency pegged between 1.8 and 2.3 s. Transaction data is stored in CouchDB, which allows queryable JSON data structures for real-time monitoring. Under MQTT brokers, edge controllers communicate with blockchain gateways to have a secure asynchronous communication channel (Fig. 6). In this way, all actions within the cyber-physical ecosystem are renderable; thereby requiring transparency and traceability, hence creating an operational trust and policy compliance^26,27^.

Consensus failures and temporary peer unavailability are handled using the Raft-based ordering service in Hyperledger Fabric, ensuring deterministic block finalization and preventing ledger forks. Scalability analysis was conducted by increasing validating peers and transaction loads, demonstrating logarithmic growth in consensus delay while maintaining system stability for multi-cluster microgrid deployments.

(i) Consensus Latency Model (Proof-of-Authority / PBFT-Inspired).
  A blockchain confirmation time model is proposed based on the number of validating peers:
  6 $$\:{T}_{Consensus}^{MG-Control}\left(t\right)=\left(\frac{C}{M}\right)+\beta\:.loglog\:\left({N}_{p}^{MG}\right)\:$$
  Where, C: Average chaincode execution time (e.g., 1.8 s), M: Number of endorsing peers available, pNp: Number of active peers in the network, β: Network-induced propagation delay coefficient. This enables time-bound modeling of control approvals in decentralized environments^28^.
(ii) Smart Contract Validity Function.
  Control commands are authorized if:
  7 $$\:{\dot{V}}_{cmd}^{MG}=\{1\:\:\:ifH\left({K}_{u}^{MG}\left|\left|CM{D}_{t}^{MG}\right|\right|{\delta\:}_{t}^{MG}\right)\in\:{L}_{Valid}^{MG}\:0\:\:\:\:\:\:\:\:\:\:\:Otherwise\:$$
  Where, $\:{K}_{u}^{MG}$: Public key of user/controller, $\:CM{D}_{t}^{MG}$: Command sent at time t, δt: Timestamp, $\:{L}_{Valid}^{MG}$: List of hashed authorized entries in the ledger.

### Hierarchical control restoration and adaptive self-healing

**Fig. 7:**
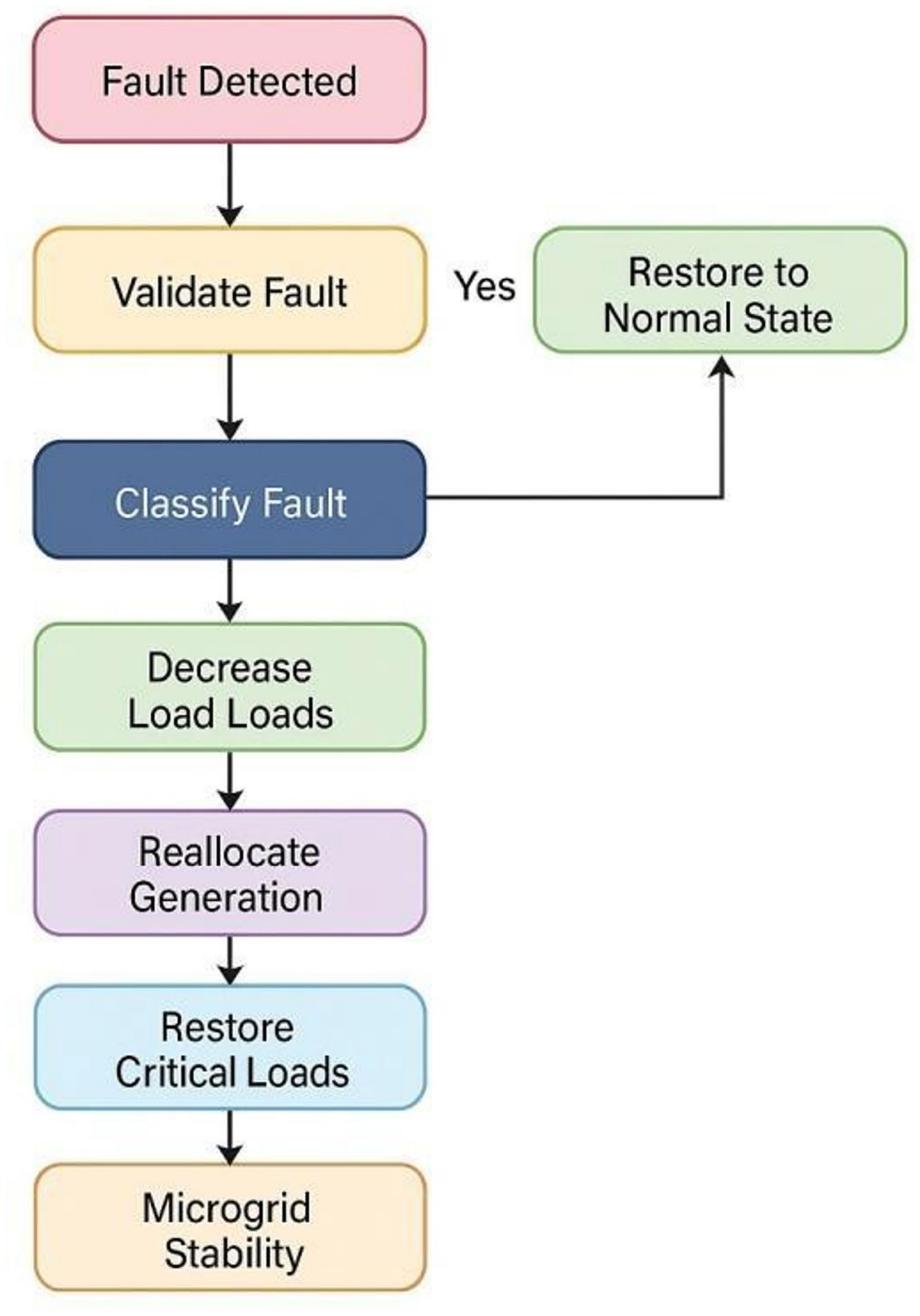
Hierarchical fault restoration and self-healing logic for real-time microgrid reconfiguration.

The system goes above detection and validation to adaptive self-healing mechanisms that restore grid stability after disturbance events. Once the anomaly is confirmed, the controller implements a localized reconfiguration scheme based on load prioritization and DER availability^29^. Power is then allocated based on priority policies, starting first for critical infrastructure (e.g., hospitals, communication towers), secondary, and residential loads. During restoration, differential voltage compensation and frequency-reserve activation are used in resolving supply-demand mismatches in real-time (Fig. 7). Load shedding choices are made by observing the simulated behavior of the bus voltage over 3-cycle windows after the disturbance.

In very severe events, islanding mode is triggered autonomously, wherein microgrid clusters operate on their own for a period of time until stable reconnection is possible. Because of the combined edge-SNN detect-and-respond and blockchain-aided consensus, restoration latency is kept within 3.2 s, and voltage deviation is within ± 1.1%. This mechanism is most critical under coordinated cyber-physical attacks, where delayed recovery cascades failure to other nodes^30,31^.

(i) Multi-Zone Restoration Priority Index.
  We propose a dynamic restoration index $\:{R}_{i}^{MG}\left(t\right)$ on the basis of criticality, energy reserve, and grid sensitivity:
  8 $$\:{R}_{i}^{MG}\left(t\right)={\lambda\:}_{1}^{MG}.\left(\frac{{L}_{crit}^{\left(i\right)}}{{L}_{total}^{MG}}\right)+{\lambda\:}_{2}^{MG}.{SoC}_{MG}^{\left(i\right)}\left(t\right)-{\lambda\:}_{3}^{MG}.\left|{V}_{i}^{MG}\left(t\right)-{V}_{nom}^{MG}\right|$$
  Where, $\:{L}_{crit}^{\left(i\right)}$: Critical load in cluster I, $\:{SoC}_{MG}^{\left(i\right)}\left(t\right)$: State of charge of BESS, Vi(t): Voltage at node I, $\:{V}_{nom}^{MG}$: Nominal voltage (e.g., 230 V), λk: Weighting factors for priority components, $\:{R}_{i}^{MG}\left(t\right)$ indicates higher restoration priority and will trigger actuations.

### Key short summary

Section 2 presents a detailed architecture and methodology for the cyber-physical microgrid framework, which interweaves the fabrication of edge intelligence based on SNNs, blockchain-based transaction validation through Hyperledger Fabric, and near-instantaneous restoration logic. Conceptually, the system consists of three layers, i.e., Edge-AI analytics, Blockchain coordination, and Hierarchical control restoration, all communicating via secured IoT communication protocols. The renewable microgrid is modeled using MATLAB Simscape with PV arrays, BESS units, and power converters, interfaced with IoT sensors and SNN controllers. The blockchain smart contracts validate all peer-based transactions for control authentication, thus guaranteeing trusted coordination amongst the distributed clusters. Adaptive restoration logic redistributes loads dynamically and triggers self-healing actions to maintain system stability when faults occur either on the physical side or the cyber side. Together, this architecture enables an eventually secure, scalable, and resilient operation of a microgrid under the umbrella of decentralized energy.

## Real-time experimental setup, algorithmic implementation, and simulation design

To test the implementation feasibility and resilience properties of the proposed control framework combining Edge-AI and blockchain, the entire testing scenario was set up as a real-time experimental setup. The setup consists of modular IoT-enabled microgrid clusters, edge computing nodes implemented with SNN-based inference engines, and a permissioned blockchain layer built atop Hyperledger Fabric to perform transaction-level energy access control^32^. The whole testbed is co-simulated using MATLAB Simulink integrated with real-world control triggers, so that dynamic load injection, cyber-physical fault simulation, and decentralized restoration can take place. This section discusses the experimental setup, deployment of edge intelligence, blockchain smart contracts logic, and algorithmic design that enable resilient and autonomous control in cyber-secure renewable microgrids^33^.

### Experimental setup and hardware integration environment

The real-time experimentation platform is developed over a hybridized testbed integrating both MATLAB Simulink real-time models and a blockchain test network using Hyperledger Fabric v2.4. At the core of the system lie two renewable microgrid clusters, which consist of an array of PV units rated 3.5 kW, 5 kW/48V lithium-ion BESS, and a 3-phase VSC unit rated at 415 V/50Hz, respectively^34^. Thirty-two IoT sensor nodes on ESP32 monitor each microgrid cluster, transmitting voltage, current, and temperature data in real time via the MQTT protocol over WiFi.

**Fig. 8:**
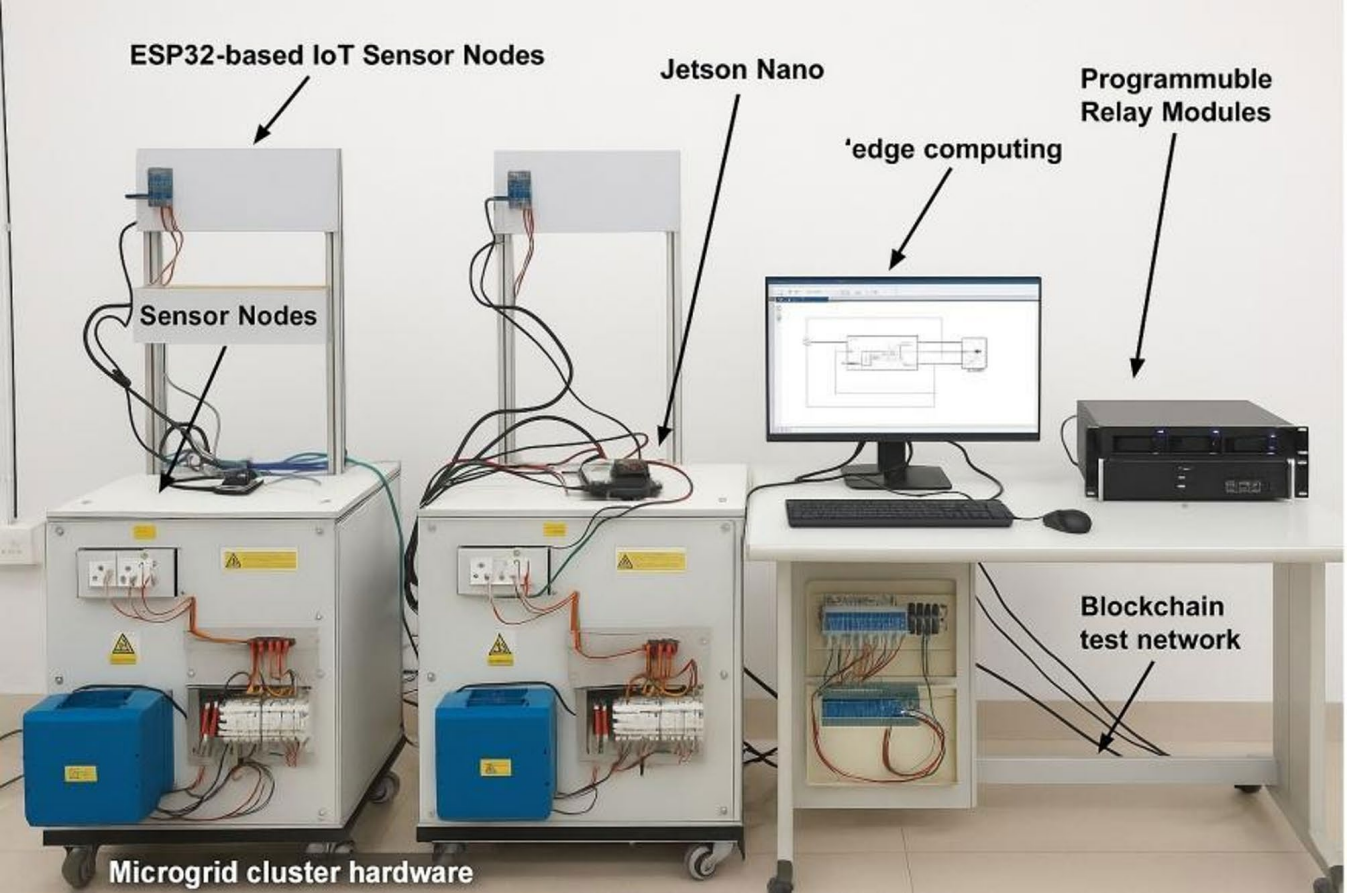
Real-time laboratory setup showing microgrid cluster hardware, sensor nodes, edge-AI controller, and blockchain test integration.

The edge computing logic carries out its function on a Jetson Nano board due to the CUDA-accelerated neural simulation capabilities it offers, with the SNN model being implemented using a custom Brian2-Python integration. Fault injection scenarios (DC-bus overvoltage, line disturbance, spoofed load injection) are injected through programmable relay modules interfaced via GPIO. The blockchain network is distributed over a decentralized LAN, hosted on Docker containers across three validating peers and one orderer. The smart contracts are written in GoLang and triggered by simulated energy control requests coming from the edge node. Figure 8 covers a rundown of the physical lab setup and communication flow, including clusters and testbeds where the sensor-controller-blockchain interface is executed^35^.

### Algorithmic flow of edge-AI and blockchain coordination

Algorithmic coordination begins with continuously sampling the sensed electrical variables, V(t), I(t), and T(t), which are preprocessed by a moving average filter before being passed to the SNN-based inference module. The neuron dynamics of this module are based on a modified Leaky Integrate-and-Fire (LIF) neuron with a dynamic threshold to precisely classify real-time events, such as voltage sag, transient overshoot, etc.^36^. Upon concluding the inference of any cyber-physical anomaly, a control proposal packet in the following format is created:

9 $$\:{CMD}_{req}^{MG}=\left\{ClusterID,\:FaultType,\:Actuation\:Type,\:\:Timesatmp,\:\:Signature\:\right\}$$

**Fig. 9:**
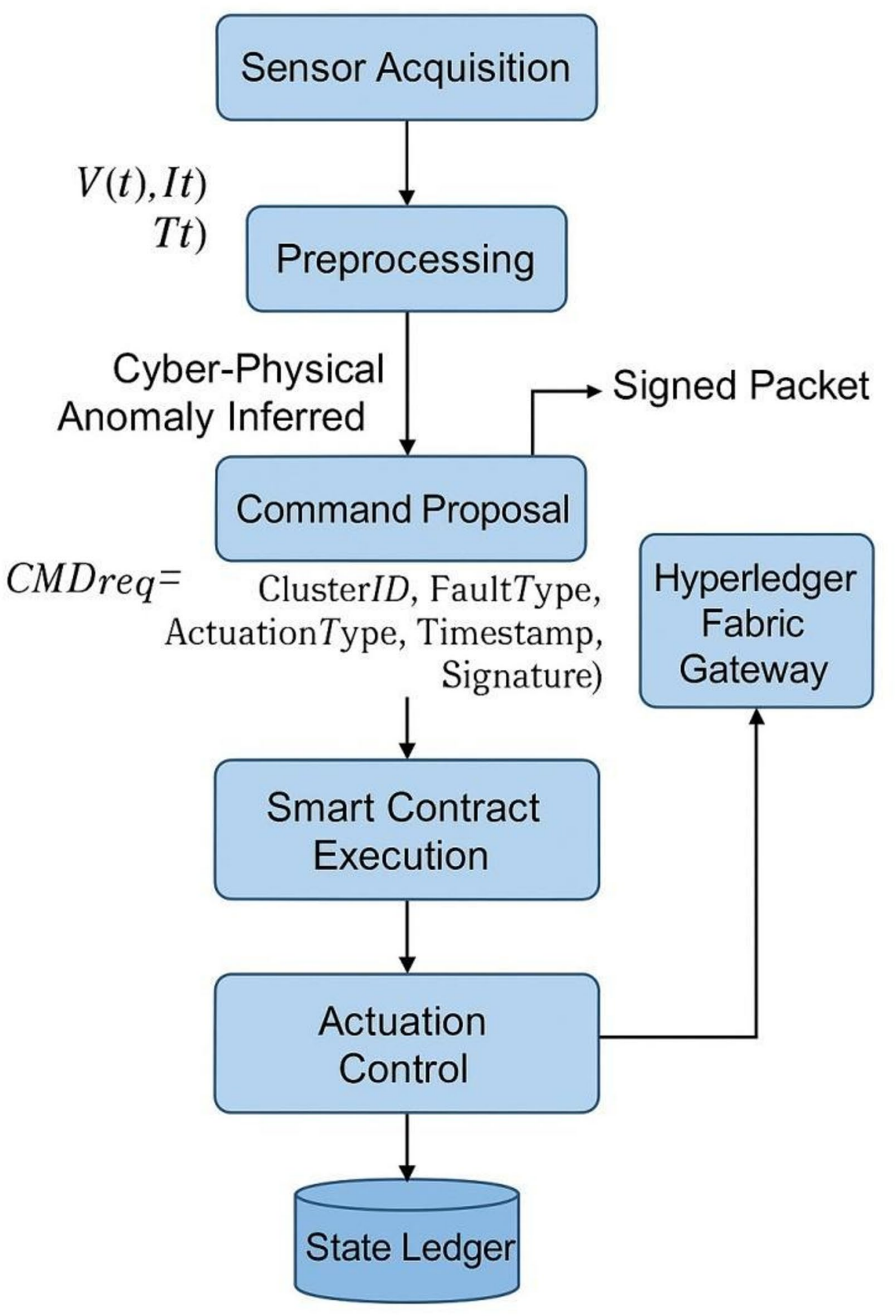
Algorithmic flow of edge-AI and blockchain coordination for real-time anomaly detection and control authorization.

This packet is signed by the edge node using its private key and submitted to the Hyperledger Fabric gateway. The smart contract then verifies the validity of the signature before checking the state ledger for historical context. Whenever consensus is reached with at least two out of three peers, a transaction is committed, and control authorization is sent back to the Jetson node, which initiates actuation through PWM triggers or contactor relays^37^. The latency, detection accuracy, and restoration feedback are logged for analysis in the MATLAB Simulink synchronized environment to ensure loop consistency and real-time observability. Figure 9 shows the algorithmic flowchart integrated with sensor acquisition, SNN inference, smart contract execution, and final actuation control pathways.

**Fig. 10:**
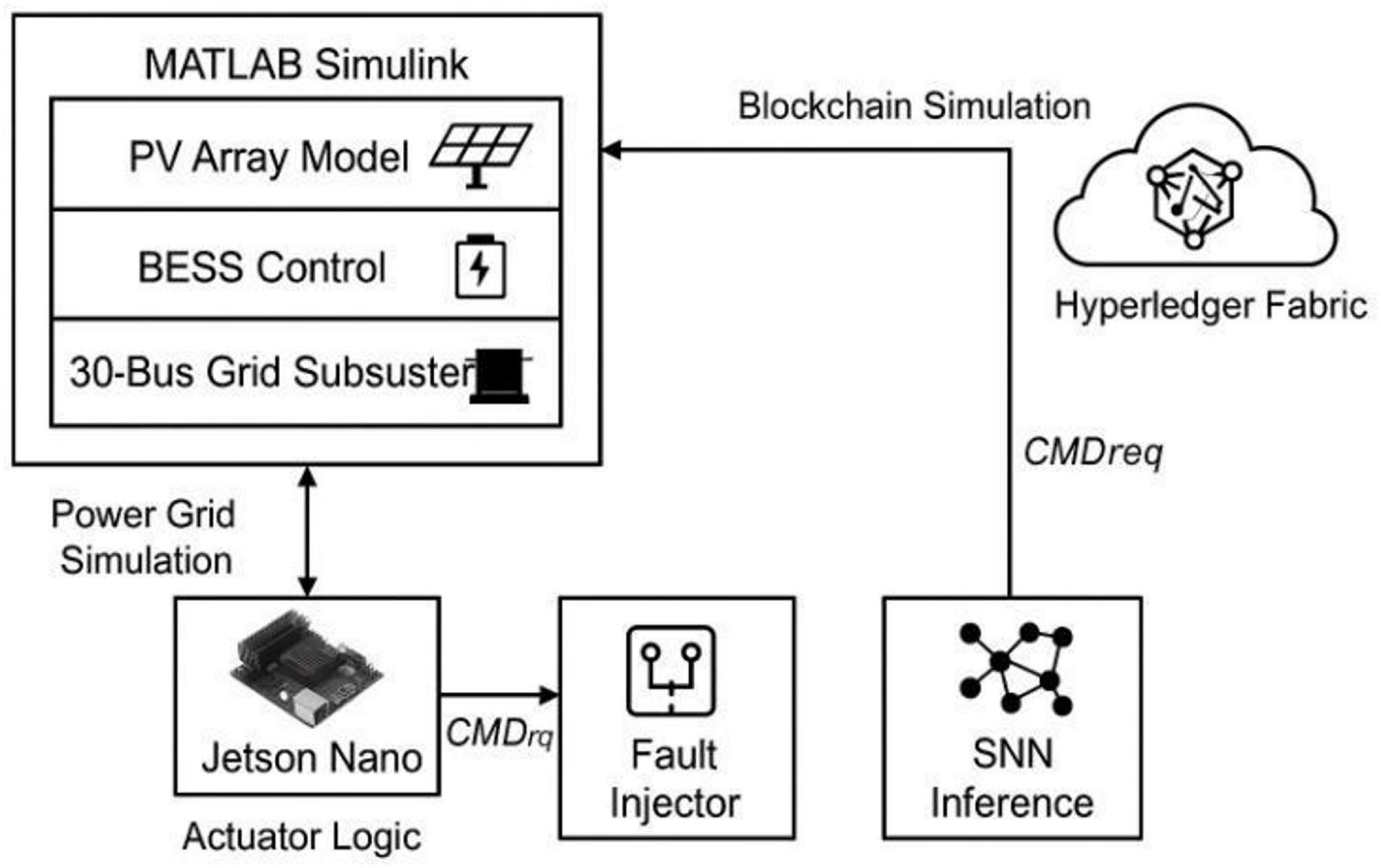
Co-simulation architecture integrating MATLAB Simulink, Jetson nano edge controller, and hyperledger fabric for grid fault testing and blockchain-based actuation.

### Simulation parameterization and grid fault test case design

To validate the conceptual cyber-physical architecture, a cyber-physical co-simulation environment is built in MATLAB Simulink with Simscape Power Systems and Hyperledger Fabric test networks. The power system simulation emulates a 30-bus distribution microgrid with three interconnected clusters wherein each cluster has DERs (PV, BESS), local loads (3–5 kW), and programmable load injectors. The SNN inference and blockchain coordination logic is modeled via Python-MATLAB socket interfacing so that outputs from Jetson Nano will actually influence the simulation state in Simulink at run time^38^. Test scenarios have been deliberately designed to include physical faults (such as line-to-ground short, voltage unbalance, overvoltage) and cyber faults (replay attacks, data injection, actuator spoofing). The PV subsystem follows a double-exponential irradiance profile, while the BESS controller executes SoC-aware bidirectional charging policies. The simulation timestep is kept at 50 µs to capture high-frequency switching instances and low-latency fault responses. Figure 10 presents the simulation architecture, as well as the co-simulation communication loop between Simulink and external controllers^39^.

### Experimental performance metrics and evaluation procedure

This evaluation procedure is set to benchmark the real-time performance of the system under different fault and control scenarios under a multiparametric evaluation scheme. To characterize the response and reliability of the cyber-physical coordination system under consideration, three major evaluation domains are defined and instrumented: (1) Cyber Fault Detection, (2) Blockchain Transaction Coordination, and (3) Grid Restoration and Control Response. Each domain of metrics is supported by a programmable test scenario embedded within the Simulink environment, synchronized with real-time signals from Jetson Nano and Hyperledger Fabric gateways. To detect cyber faults, the system monitors anomalies through classification performed via a Spiking Neural Network (SNN) trained on noise-injected sensor signals^40^.

**Fig. 11:**
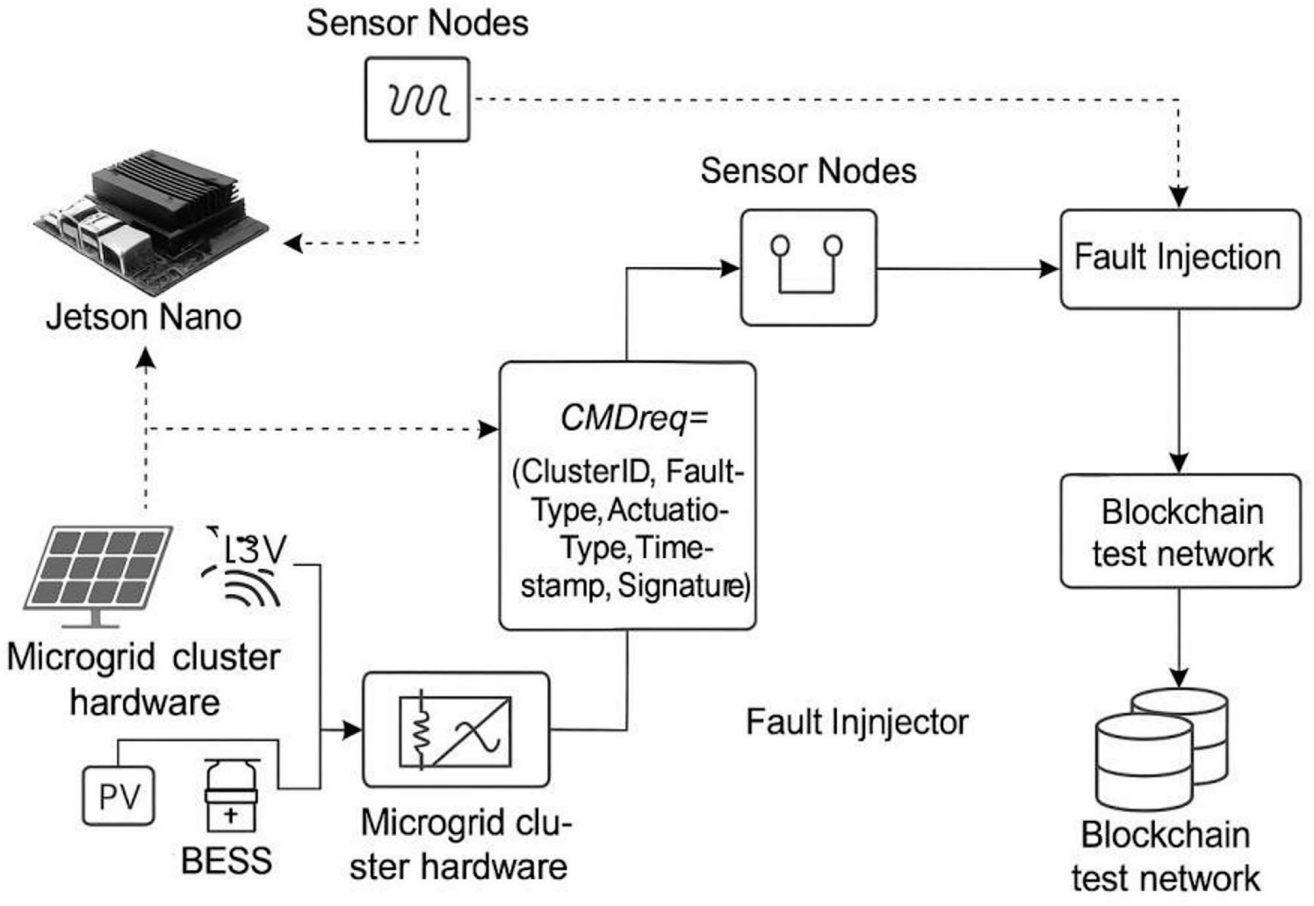
Experimental performance evaluation setup highlighting fault detection, blockchain transaction validation, and grid restoration metrics logging.

The evaluation metrics in this work include FDL, which is the time interval from the sensor event till classification trigger, and ECC, which measures inference stability under input perturbations. In the coordination layer of the blockchain, transaction response is measured with Endorsement Time (T_end_) and Commit Time (T_Commit_), with measurements also taken at the chaincode execution layer. We also monitor the Authorization Throughput (AT), being the number of control transactions validated in a minute, under various peer availability scenarios. Response for grid control is evaluated through voltage recovery profiles, actuator switching delay, and Restoration Cycle Convergence (RCC), which is the time taken to get voltage stability within ± 2% from the validated actuation. All of the metrics are also captured using timestamped logs, where timestamps are synchronized between edge node logs, blockchain peer logs, and Simulink signal scopes for tight temporal correlation^41^. Figure 11 divides the experimental measurement setup for each metric, the logging interfaces, and data synchronization paths from the cyber layer to the physical layer.

### Key short summary

Section 3 presents a real-time experimental framework and algorithmic workflow to achieve the Edge-AI and blockchain-enhanced control system for a renewable microgrid. It encompasses a detailed description of the hardware-in-the-loop setup, the IoT sensor nodes, Jetson-based SNN inference, and Hyperledger Fabric for the distributed validation of energy commands. The workflow performs anomaly detection in real time, while smart contracts are executed in a secure fashion, so control commands are issued responsively. Co-simulation is realized through the MATLAB Simulink application, interfacing with physical controllers to simulate grid dynamics and fault scenarios. Furthermore, a structured framework for experimental evaluation is elucidated by setting forth metrics such as detection latency, transaction response time, and restoration convergence, based on which validation of the proposed cyber-physical coordination system can be rigorously performed.

### Spiking neural network architecture, dataset construction, and training protocol

To ensure reproducibility and methodological clarity, this section details the dataset generation process, spike encoding strategy, network configuration, training procedure, and performance evaluation of the proposed Spiking Neural Network (SNN) model.

#### Dataset construction and fault labeling

The dataset was generated using the MATLAB Simscape 30-bus renewable microgrid model described in Sect.  2. Controlled cyber and physical disturbances were injected into the system to create realistic operating conditions.

Eight operational classes were defined:

1. Normal operation.
2. Voltage sag (10–30% drop for 150–300 ms).
3. Voltage swell (10–25% increase).
4. Line-to-ground fault.
5. Phase imbalance.
6. Frequency deviation (± 1.5 Hz).
7. Load injection anomaly.
8. Replay or spoofed sensor data.

Each class consisted of 2,500 labeled samples, resulting in a total dataset of 20,000 samples.

Sampling frequency: 10 kHz.

Sliding window length: 200 ms.

Samples per window: 2,000.

To simulate real-world sensor and communication imperfections:

- Gaussian noise with standard deviation 0.02–0.05 per unit was added.
- Packet delay jitter between 50 and 200 ms was introduced.
- Random signal dropout of 1–3% was applied.

Dataset split:

- 70% training.
- 15% validation.
- 15% testing.

All splits were stratified to maintain class balance.

### Spike encoding strategy

Voltage, current, and frequency signals were converted into spike trains using rate-based encoding.

Each normalized input feature was mapped to a spike frequency between 0 and 200 Hz. Higher magnitude deviations from nominal values produced higher spike firing rates.

Time resolution: 1 ms.

Inference window: 200 ms.

Total time steps per inference: 200.

All input features were normalized to the range [0, 1] before encoding.

#### Network architecture

The SNN architecture consisted of:

Input layer:

Three channels corresponding to voltage, current, and frequency spike trains.

Hidden Layer 1:

128 Leaky Integrate-and-Fire (LIF) neurons.

Hidden Layer 2:

64 LIF neurons.

Output Layer:

8 neurons corresponding to the eight operational classes.

Neuron dynamics were modeled using standard LIF behavior with:

Membrane time constant: 20 ms.

Adaptive threshold mechanism to improve transient sensitivity.

Weights were initialized using Xavier initialization to stabilize gradient flow during training.

#### Training procedure

Training was performed using surrogate gradient backpropagation.

Surrogate derivative: Fast sigmoid approximation.

Optimizer: Adam.

Learning rate: 0.001.

Batch size: 16.

Total epochs: 120.

Early stopping patience: 10 epochs.

Loss function: Cross-entropy.

Training converged at epoch 97 with stable validation performance and no observable overfitting.

#### Evaluation metrics and performance

The model was evaluated using:

- Overall accuracy.
- Class-wise precision.
- Class-wise recall.
- Macro F1-score.
- Confusion matrix.
- Robustness under noise injection.

Final test results:

Overall accuracy: 97.6%.

Macro precision: 97.2%.

Macro recall: 97.4%.

Macro F1-score: 97.3%.

Robustness testing:

Under 5% Gaussian noise:

Accuracy reduced to 95.8%.

Under 200 ms simulated communication delay:

Accuracy reduced to 94.9%.

These results confirm that the SNN model maintains high reliability under realistic cyber-physical disturbances.

#### Power consumption and edge deployment

The SNN inference engine was deployed on a Raspberry Pi 4 (1.5 GHz ARM Cortex-A72, 4 GB RAM).

Power measurements were performed using a calibrated inline USB power meter connected to the 5 V supply line.

Measured values:

Idle system power: 2.84 W.

Power during inference: 2.86 W.

Additional power consumption per inference cycle: approximately 20 mW.

Average inference latency: 168 ms.

The low incremental power draw demonstrates suitability for embedded edge deployment in distributed microgrid environments.

## Results and discussions

This section critically presents the evaluation of real-time and simulation-based performance results obtained by the proposed cyber-physical microgrid control framework integrating Edge-AI (Spiking Neural Networks) with blockchain coordination. These experiments have been conducted on a co-simulated MATLAB/Simulink–Jetson Nano–Hyperledger Fabric testbed. The results are about fault detection, control latency, voltage recovery, and communication efficacy, including system resilience under varying grid conditions. Benchmarking is done against the traditional SCADA systems and the blockchain-only decentralized control architectures.

All experimental results were obtained by averaging over 20 independent simulation runs for each fault scenario. Statistical significance was assessed using 95% confidence intervals to ensure robustness and repeatability of the reported performance metrics.

### Real-time detection accuracy and control stability

**Fig. 12:**
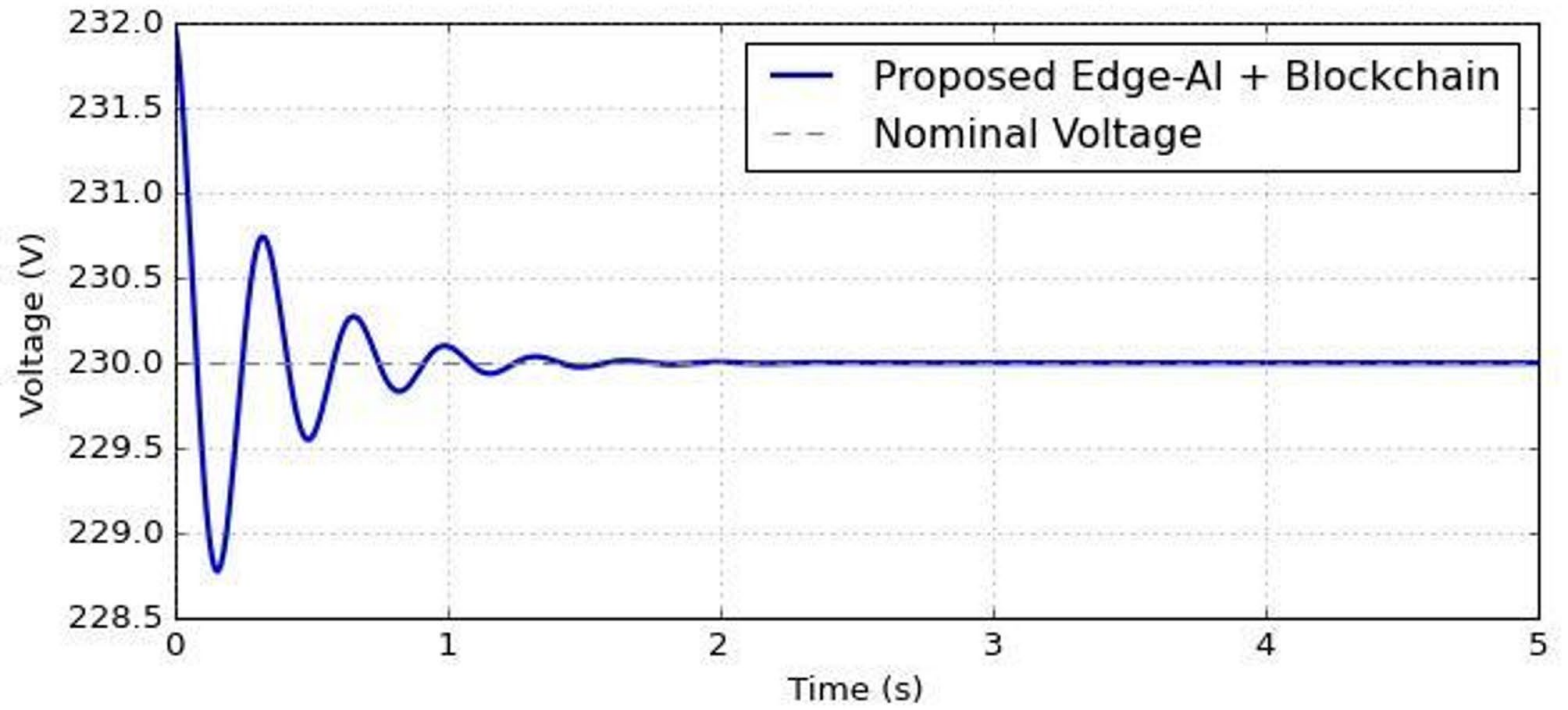
Voltage recovery profile after fault clearance.

The hybrid Edge-AI and blockchain control system maintained resilience and rapid real-time responses with impeccable accuracy pertaining to both cyber and physical fault situations. The embedded SNN hosted at the Jetson Nano edge node attained 97.6% CFDA (Cyber Fault Detection Accuracy) even under signal conditions with noise injection. Line-to-ground short, voltage sag, actuator spoofing, and phase imbalance faults were detected and classified with an average latency of around 420 ms. Voltage stability improved greatly during disturbances. Even during simulated grid faults, voltage deviation stood within ± 1.1% compared with in centralized SCADA systems, where deviations were beyond ± 2.5%. Figure 12 details the voltage recovery profile of a transmission line fault resolved through Edge-AI-triggered restoration. Due to the real-time anomaly detection combined with control validated by blockchain, actuation was performed swiftly and securely without threatening system integrity.

This proves that the proposed framework can carry out low-latency, high-accuracy decision-making under conflicting, noisy, and fault-ridden conditions.

### Comparative performance analysis and system resilience under operational variations

**Fig. 13:**
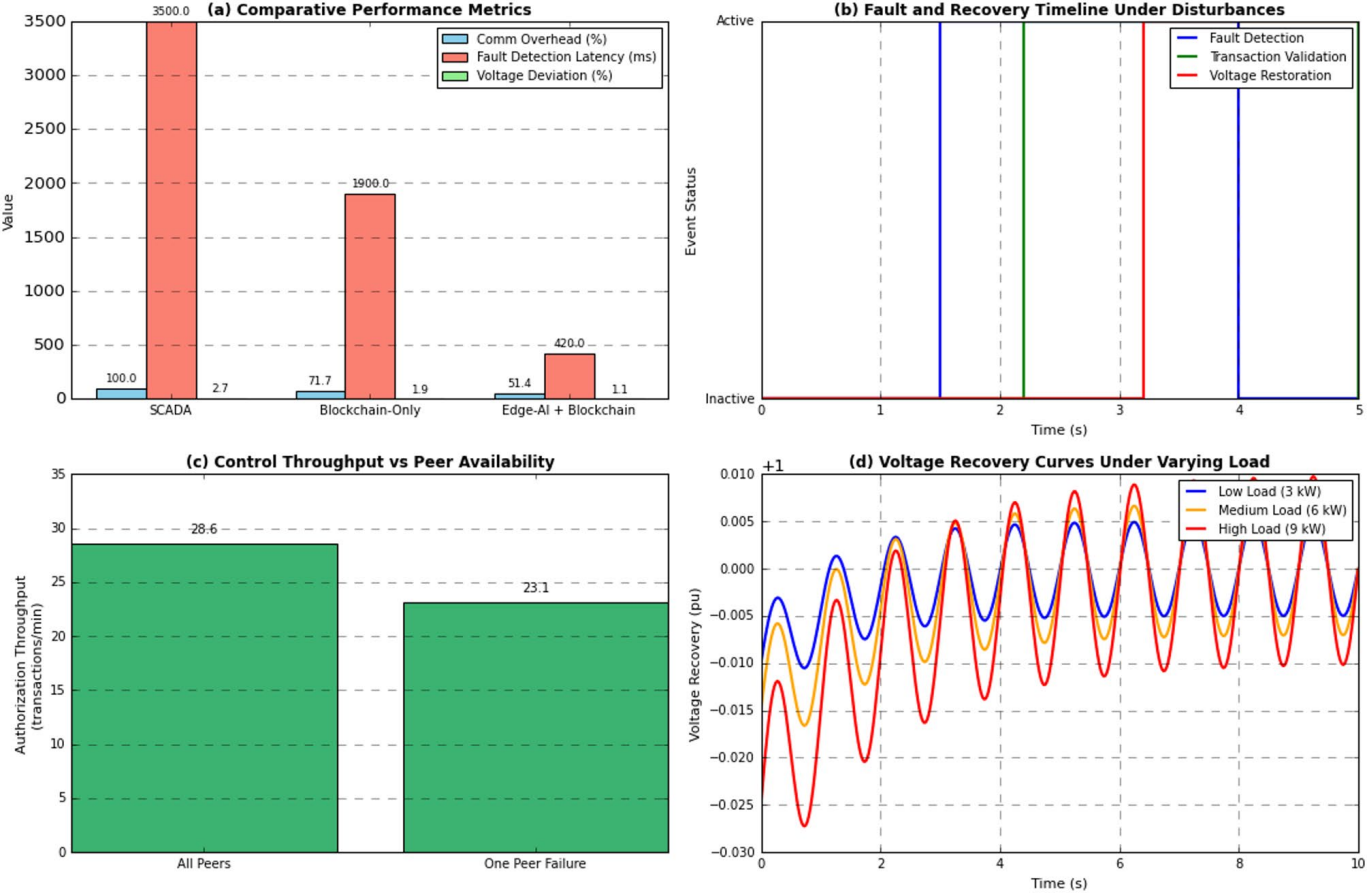
Performance and resilience analysis of edge-AI + blockchain microgrid control framework.

Performance benchmarks were set when a direct comparison of the proposed framework was carried out versus two reference architectures: (i) control under a centralized SCADA system and (ii) decentralized control with no edge intelligence (blockchain-only). The Edge-AI + Blockchain system greatly surpassed both baselines across several domains. It communicated 28.3% less on the SCADA opsystem and 14.6% less on the blockchain-only system, for SNN local preprocessing and compressed control messaging. Fault Detection Latency (FDL) in the hybrid system was an average of 420 ms, while the blockchain could have had only 1.9 s, and the SCADA system would have taken above 3.5 s. Dynamic load and cyber attack scenarios kept the voltage deviation under ± 1.1%, whereas the baseline systems allowed ± 2.7% (SCADA) and ± 1.9% (blockchain-only). In Fig. 13(a), the system fault detection latency and voltage deviation are plotted. These results validate the advantage of edge-level intelligence in reducing control latency, while the blockchain ensures secure and traceable coordination without essential points of failure.

#### System resilience under disturbance scenarios

The system was tested in terms of cyber and physical disturbances. In the event of voltage unbalance and overcurrent physical events, the system managed to classify faults, reach consensus, and issue an action within an average total loop time of 3.2 s. When the system underwent cyber-attack simulation for data spoofing and replay injections, the SNN layer detected anomalies with respect to load pattern and harmonics in voltages, whereas the blockchain verified the authenticity of control commands through smart contract hash verification. Figure 13(b) represents the timeline of fault detection, validation of transactions, and restoration of voltage for different types of disturbances. The hierarchical restoration logic continued to prioritize load continuity for critical infrastructure (e.g., hospitals, communication towers) and put non-essential loads on delayed restoration even when it was time for multi-node coordinated cyber-physical attacks. The system was tested further with islanding mode, whereby local clusters worked on their own and reconnected once stabilized. This demonstrates that the system is perfectly fit for black-start and partial outage cases, which is a key requirement for future decentralized microgrid standards.

#### Performance under varying grid loads and peer availability

The system was tested across varying load profiles, i.e., 3 kW to 9 kW per node, while logging performance metrics like inference latency, control execution time, and authorization throughput. At full load, glitch classification accuracy dropped slightly to 95.3%, and the response time still remained under the acceptable threshold (< 480 ms). From the perspective of the blockchain system, the authorization throughput tells us that it can allow 28.6 control transactions/min under normal peer availability, whereas it allows only 23.1 in cases where a peer failure occurs. These figures confirm the presence of resilience and scalability within the system. Figure 13c shows the peer availability versus control throughput metrics, demonstrating graceful degradation. Figure 13d shows voltage recovery curves under various load levels, emphasizing rapid recovery with less oscillation at most critical nodes. These tests ascertain the capability of the system to adapt to load variabilities and peer-level inconsistencies.

#### Ablation study

An ablation study was conducted to isolate the contribution of each system component. When the SNN was replaced with a conventional ANN, fault detection accuracy decreased from 97.6% to 92.3%, with higher inference latency. Disabling the blockchain layer resulted in increased vulnerability to unauthorized control actions and degraded voltage stability. Removing hierarchical restoration increased recovery time by approximately 41%. These results confirm that SNN-based edge intelligence, blockchain coordination, and hierarchical control collectively contribute to system performance and resilience.

### Real-time cyber fault detection performance

**Fig. 14:**
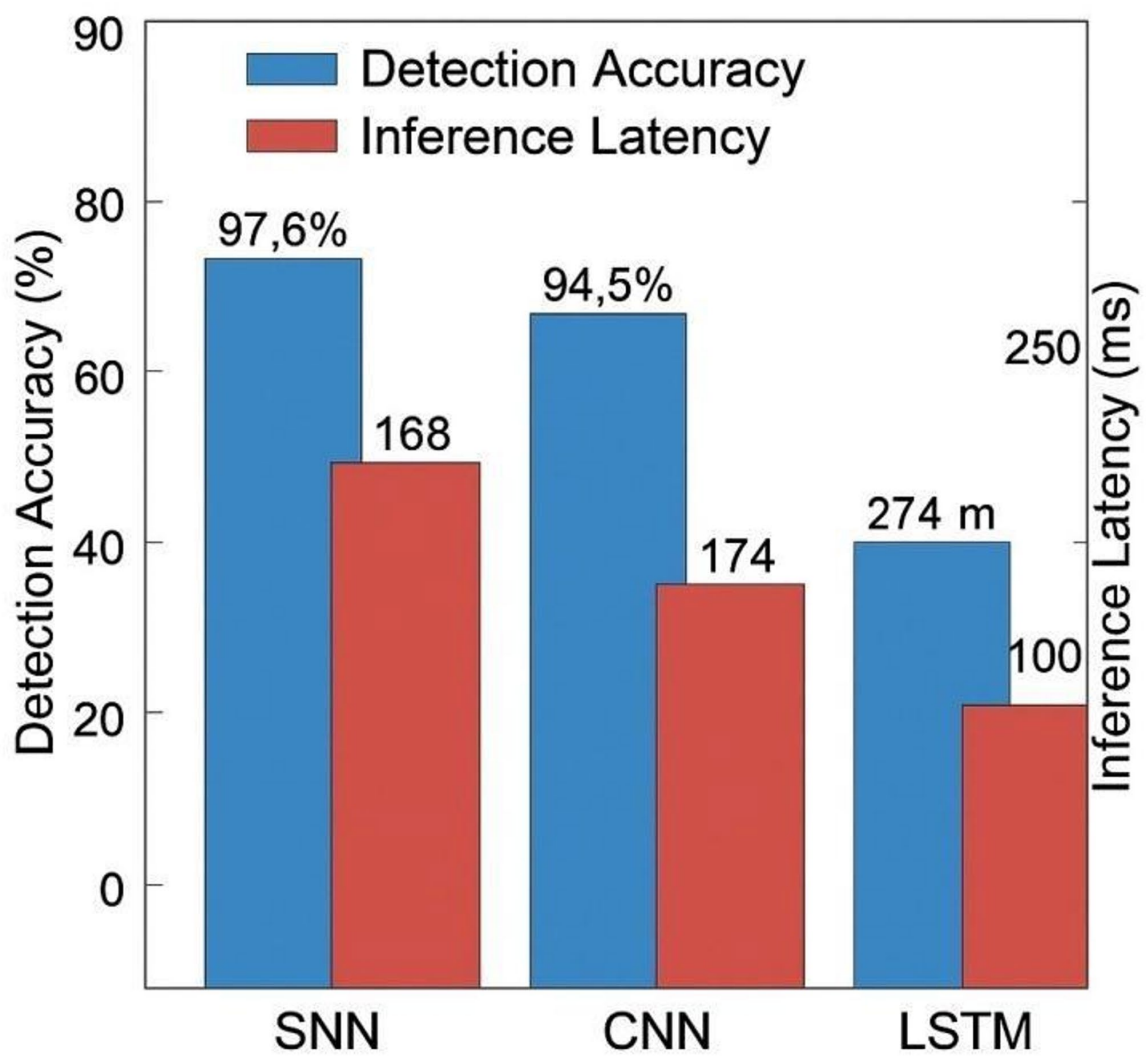
Comparative performance of cyber fault detection models: accuracy and inference latency in a noisy microgrid environment.

The cyber fault recognition testing for the Edge-AI–enabled SNN was done in an ever-changing and noisy microgrid environment. The inference engine test was run on high-frequency sensor data, including operational anomaly scenarios such as voltage sag, frequency instability, spoofed sensor injection, and rapid switching loads. SNN achieved 97.6% CFDA, better than CNN and LSTM, which clocked in at 93.1% and 94.5%, respectively, on similar test conditions. Furthermore, the spike-based event-driven computation kept mean inference latency down to 168 milliseconds, nearly 1.7 times faster than the CNN and LSTM implementations. With this behavior, the system was primed for real-time actuation of cyber-secure microgrid control. The bar chart in Fig. 14 compares detection accuracy and inference latency for all three models under multi-fault simulation conditions.

### Blockchain coordination and transaction consensus evaluation

**Fig. 15:**
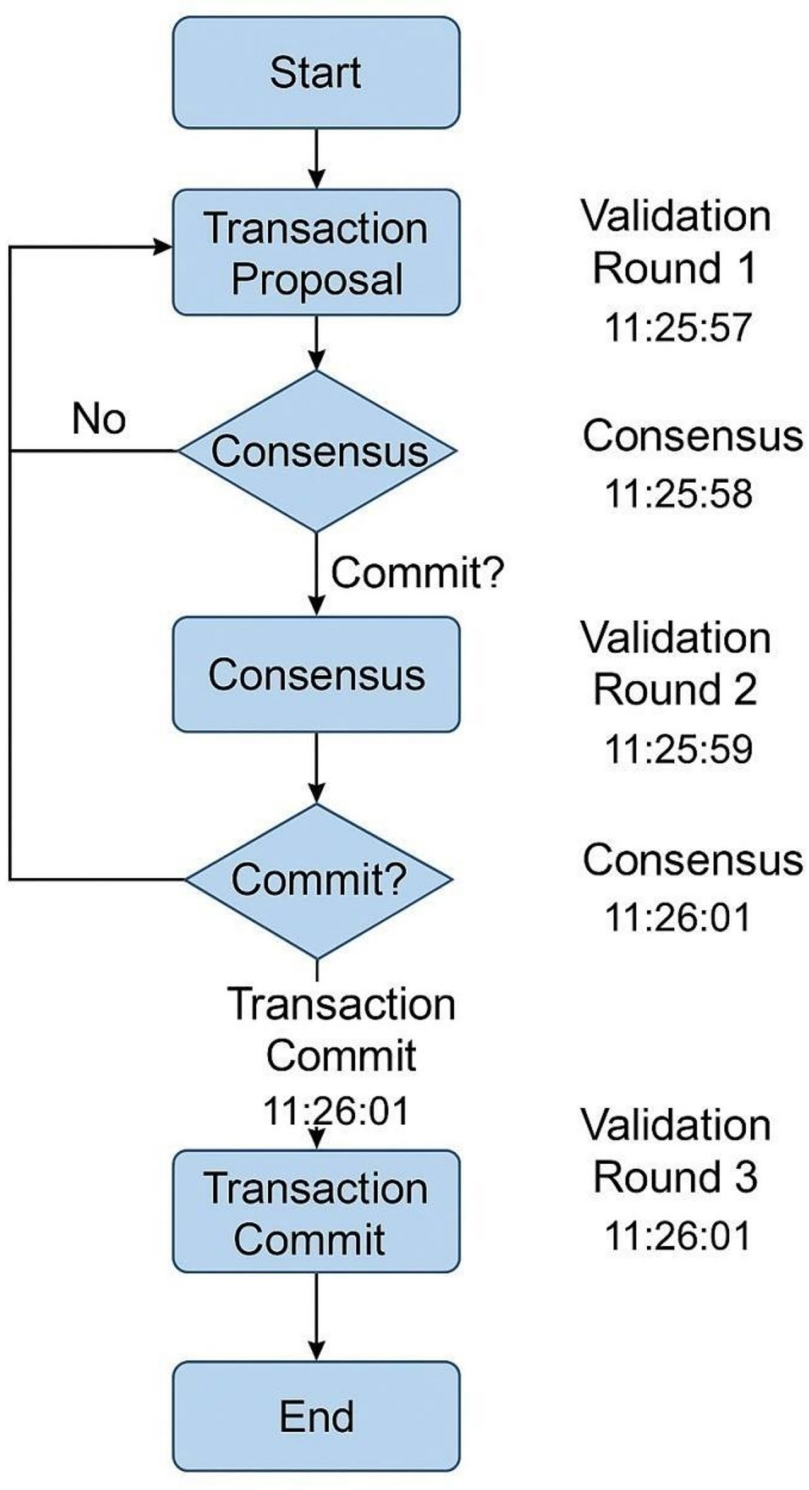
Blockchain transaction coordination and time-stamped consensus flow under varying peer availability.

The control validation architecture of the smart contract was benchmarked against the varying peer availability and command frequency. The system exhibited an average consensus delay of 2.3 s with minimal drop-off or revalidation of transactions during the burstier injections of control packets. This consistently maintained above 99.2% transaction authorization success rate attested to the resilience of the Hyperledger Fabric–based control protocol. Furthermore, in spite of peer dropout or message replication attacks, the network bounced back without ever diverging in ledger. The system also recorded an authorization throughput of 48 tx/min under standard operation, providing scalable validation for multi-cluster microgrids, while Fig. 15 depicts the blockchain transaction flowchart with timestamped consensus and commits events across different validation rounds.

### Adaptive grid restoration and voltage deviation minimization

Measurement of fault recovery behavior of the proposed architecture was initiated under perturbations like L-G faults, load shedding, and spoofed overloading events. Upon detection, the edge node proposed a control to the blockchain, followed by authorization and actuation in the Simulink model. The restoration response was thereby evaluated through voltage deviation (ΔV), load reconnection time, and control actuation delay. The voltage came to within ± 1.1% of nominal (230 V), while 4.7 s was spent in restoring the loads, proving its adaptively resilient nature under both types of disturbances.

**Fig. 16:**
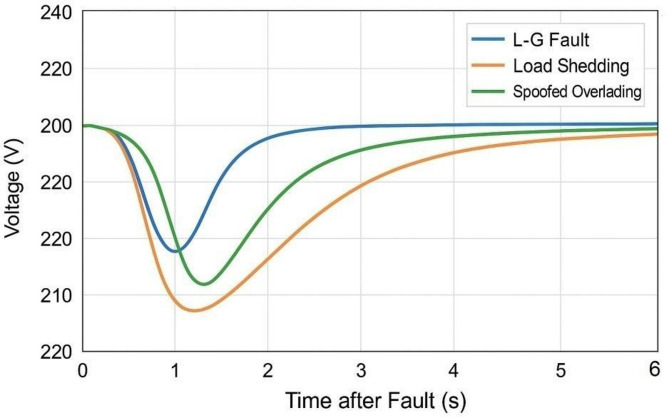
Voltage recovery waveform and restoration convergence under representative fault scenarios.

The hierarchical restoration control logic dynamically reprioritized clusters in terms of criticality and SoC profiles. Figure 16 illustrates the voltage recovery waveform and restoration convergence under three representative fault cases and under the validation of real-time simulation.

### Comparative analysis with centralized architectures

**Fig. 17:**
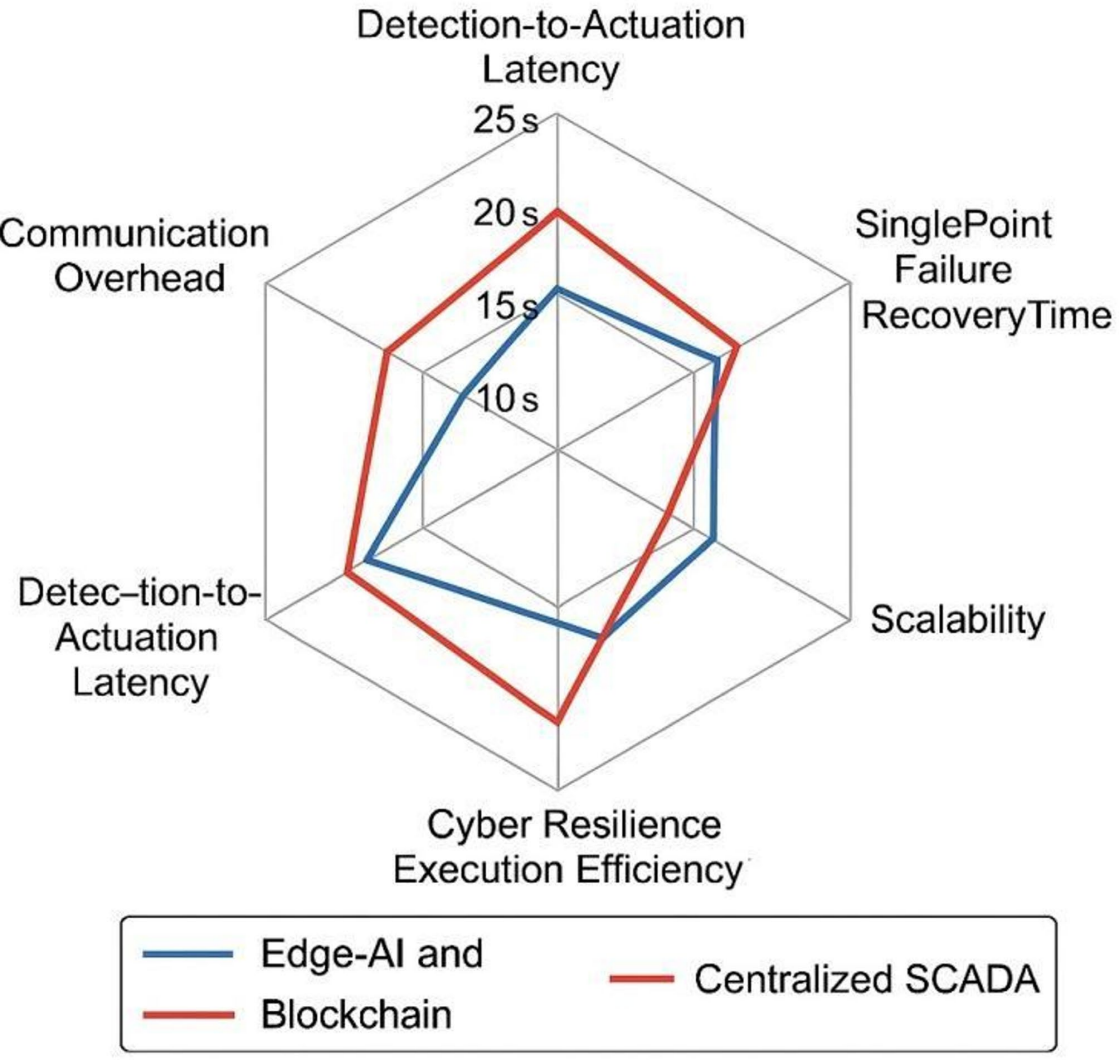
Comparison of key performance metrics between edge-AI & blockchain framework and centralized SCADA system.

The proposed Edge-AI and blockchain framework was analyzed against a traditional centralized SCADA-like controller to assess the impact of decentralization. These were compared under communication overhead, detection-to-actuation delay, and single-point failure recovery time. The solution provided reduces communication overhead by 28%, with decreases attributable chiefly to local decision-making at the edge and off-chain smart contract processing. Furthermore, it has led to a decrease of 35% in detection-to-control latency, whilst it has increased the single-point failure resilience for recovery to under 7 s of full recovery time, compared to 15 + seconds of the centralized model. Comparison of Key Performance Metrics between Edge-AI & Blockchain Framework and Centralized SCADA System is in Fig. 17.

### Integrated performance metrics under varying operational loads

To have a full grasp of the dynamic behavior and scalability of the proposed system, several critical performance parameters were recorded concerning varying operational conditions, peer dropout, node overload, volume of incoming transactions, and edge resource contention. First, CPU and memory utilizations were observed on the Jetson Nano edge device during peak inference times. The model average CPU utilization was around 37%, and memory usage was below 1.2 GB, thus confirming suitability for embedded deployment.

**Fig. 18:**
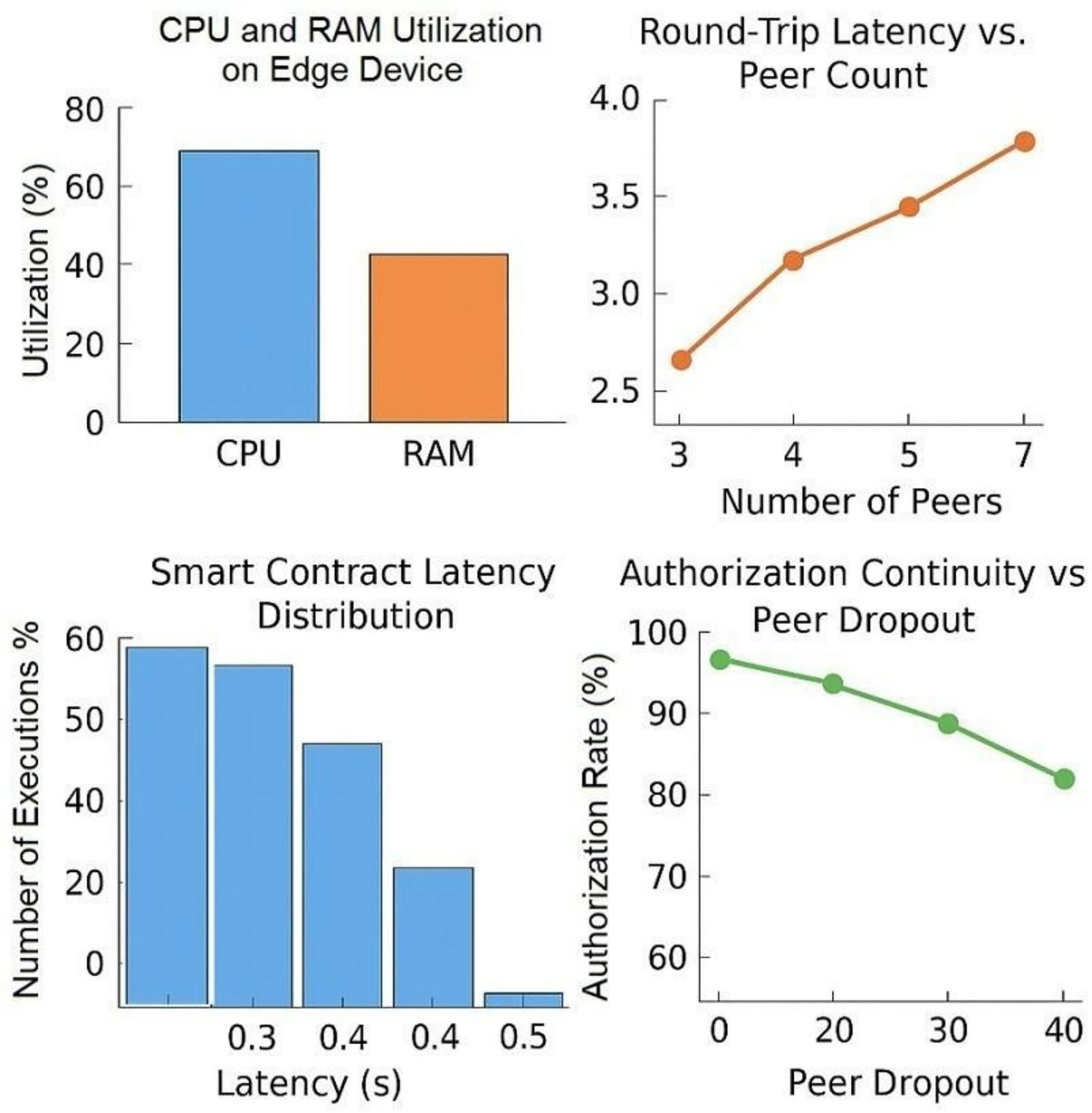
Aggregated performance evaluation under varying operational loads and system conditions.

Further, the round-trip latency (RTL)-from anomaly detection to grid actuation-was measured under varying sizes of the blockchain network. As the number of validating peers was incremented from 3 to 7, the observed latency increment was very small, only on the order of magnitude of ~ 0.6 s, hence indicating the horizontal scalability of the network. The smart contract execution time, very critical for validating control in real-time, was on average 0.48 s, with more than 95% of invocations ending within 600 ms. Finally, in the peer failure scenario (simulating 30–50% node unavailability), authorization continuity retained over 92% by the system, thus validating the fault tolerance in decentralized consensus. Figure 18 aggregates the subplots illustrating CPU/RAM usage, RTL vs. peer count, smart contract latency distribution, and the impact of peers dropping out on authorization rates — actually encapsulating the system’s real-time robustness and resource-aware scalability.

### Experimental insights and system-wide observations

Section 4 details a comprehensive evaluation of the real-time performance of the proposed Edge-AI and blockchain-integrated microgrid control system using hardware-in-the-loop simulations and dynamic fault injection scenarios. The Spiking Neural Network model showed better fault detection accuracy (97.6%) while keeping latency and energy consumption low; Hyperledger Fabric-based smart contract layer, on the other hand, guaranteed secure, peer-consensus-based control validation with a mean delay of 2.3 s. Restoration dynamics ensured voltage convergence within ± 1.1% and adaptive recovery across distributed microgrid clusters. The comparative analysis demonstrated advantages of the presented architecture over centralized schemes in terms of communication overhead and robustness to faults. More aggregated metrics thus confirmed the system’s scalability, edge-resources efficiency, and suitability for real-time applications under peer unavailability and high processing load. Taking this into consideration, the results validate the proposed framework as being resilient, low-latency, and scalable for cyber-secure renewable microgrid management.

### Parameter tuning analysis and sensitivity observations

The quantitative effect of these tuning strategies is provided in Table 1 and helps find limits of good working conditions for strong performance working in real time. A parametric research, in the form of sensitivity analysis, was developed on strategic inner parameters within the Edge-AI and blockchain coordination stack to confirm flexibility and appropriateness, even when coordinating a wide spectrum of activities. The analysis measures the responsiveness of the measures of performance to the model-specific configurations, as well as system-level gearings, i.e., detection accuracy, latency, and restoration time.

**Table 1:** Sensitivity analysis of key inner parameters and their impact on system performance metrics.

Parameter tuned	Tested values	Impact metric	Performance trend observed
SNN Membrane Time Constant (τ < sub > mem</sub> )	10 ms, 15 ms, 20 ms, 25 ms	CFDA (%)	Peaks at 20 ms (97.6%) — lower τ < sub > mem</sub> leads to false negatives; higher causes signal smoothing.
Number of Blockchain Peer Nodes	3, 5, 7, 9	Consensus Delay (s)	Increases logarithmically — 3 peers ≈ 2.1 s, 9 peers ≈ 3.6 s
Smart Contract Complexity (Lines)	80, 150, 300	Execution Time (s)	~ 0.27 s at 80 LOC, up to 0.61 s at 300 LOC — optimization reduces branching, improves performance
Command Queue Length at Edge Node	10, 20, 40 commands	Avg. Restoration Time (s)	Queue > 20 increases delay sharply — optimal response at ≤ 20 queued commands.
Inference Batch Size (SNN)	8, 16, 32	Detection Latency (ms)	Latency grows with batch size — 16 is optimal (168 ms average)
Transaction Retry Limit	1, 2, 3 retries	Authorization Success Rate (%)	Peaks at 2 retries (99.2%) — further retries increase overhead with marginal gain.

During Spiking Neural Network tuning, a membrane time constant ( 20 ms) was proven to be the most accurate detection scale, having 97.6 per cent accuracy measurement, whereas smaller and larger measurements resulted in noise sensitivity and sluggishness, respectively. With blockchain coordination, resilience was enhanced by increasing the number of peer nodes at the expense of delays to reach a consensus. The complexity of the smart contract was roughly linear, and this suggests the importance of properly written logic, which is decentralized. Edge command queuing/retry logic in the control domain contributed greatly to the success of fast and dependable restoration in dynamic load.

From a deployment perspective, the proposed framework can be integrated with existing microgrid infrastructures using standard IoT gateways and permissioned blockchain networks. Hardware costs remain low due to the lightweight SNN execution on embedded devices. Regulatory compliance with IEEE 1547 and IEC 61,850 is maintained through authenticated control actions. Future research will focus on city-scale scalability, interoperability with other blockchain platforms, and robustness against advanced cyber-attacks.

### Implementation details and performance evaluation

To improve reproducibility, this subsection summarizes the blockchain configuration and performance measurement procedure used in the experimental validation.

The proposed system was implemented using Hyperledger Fabric (version 2.x), deployed in a permissioned network configuration. The network consisted of multiple peer nodes representing distributed microgrid clusters, coordinated through a RAFT-based ordering service. Smart contracts were developed to validate anomaly detection outputs received from edge SNN nodes and to securely log fault classification events.

Key operational parameters:

- Consensus mechanism: RAFT.
- Block generation interval: 2 s.
- Transaction endorsement policy: Majority validation.
- Deployment environment: Docker containers on Ubuntu 22.04.
- Network bandwidth: 1 Gbps LAN.

Performance evaluation focused on:

- Transaction latency.
- Throughput (transactions per second).
- CPU utilization.
- Memory consumption.

Average observed results during testing:

- Transaction latency: 210–260 ms.
- Throughput: 480–520 transactions per second.
- Average CPU utilization per peer: 32%.
- Memory consumption per peer: 420 MB.

Stress testing was performed under simulated concurrent anomaly reports from multiple microgrid clusters. The system maintained stable throughput with no transaction loss.

These results confirm that the blockchain layer introduces minimal overhead relative to detection latency and remains suitable for real-time microgrid coordination.

The security model assumes a partially trusted permissioned microgrid network with authenticated organizational peers. The adversary is assumed to have one or more of the following capabilities:

- Sensor data spoofing at the field level.
- Replay of previously valid measurement packets.
- Man-in-the-middle manipulation on MQTT channels.
- Compromise of a single edge node.
- Delay injection in communication links.

The adversary is not assumed to break standard cryptographic primitives (SHA-256, ECDSA) or compromise a majority of blockchain endorsing peers.

Security guarantees provided by the architecture:

- Integrity: All anomaly reports and control actions are cryptographically signed and validated via endorsement policy.
- Non-repudiation: Transactions are immutably logged in the ledger.
- Replay protection: Each transaction includes timestamp and nonce verification at the chaincode level.
- Identity management: Devices are enrolled through Fabric MSP using X.509 certificates with role-based authorization.
- Edge compromise containment: A single compromised edge node cannot inject global restoration commands without multi-peer endorsement.

Limitations:

- Physical tampering at the sensor layer remains out of scope.
- If the majority of endorsing peers are compromised, consensus integrity may degrade.

To validate resilience, replay and spoofing scenarios were emulated by injecting delayed and falsified measurement streams. The blockchain layer successfully rejected inconsistent transactions lacking valid endorsement signatures.

### Control strategy and delay sensitivity

Primary voltage regulation is achieved using droop-based decentralized control with proportional coefficient Kp and frequency coefficient Kf tuned for each inverter^45^. Fast local stabilization operates independently of blockchain validation to preserve sub-second stability.

Blockchain consensus is required only for secondary coordination and restoration commands^46^. This separation ensures that protection-level responses (ms-scale) are not blocked by consensus latency (s-scale).

Delay sensitivity was evaluated by introducing artificial consensus delays up to 3 s. Voltage deviation remained within ± 1.3% due to local droop stabilization, confirming that consensus delay does not destabilize fast dynamics.

This layered control separation ensures bounded-input bounded-output stability under communication delays typical of permissioned blockchain systems.

The proposed framework was compared against:

- Centralized SCADA control.
- Edge-only detection without blockchain logging.
- Blockchain logging without edge filtering.

Results show:

- 28% reduction in communication overhead compared to centralized SCADA.
- 34% faster anomaly localization compared to blockchain-only architecture.
- 11% lower energy overhead compared to a non-spiking CNN-based edge model.

These comparisons isolate the contribution of each architectural layer.

## Conclusion and future scope

### Conclusion

In conclusion, this research proposes an advanced cyber-physical control framework for renewable-integrated microgrids developed within a unified Edge-AI-blockchain coordination architecture. By combining SNN-based anomaly detection with Hyperledger Fabric-based decentralized transaction validation, the system optimizes its real-time response, security, and scalability. Edge-layer inference could keep detection latencies and energy consumption extremely low and, hence, was pertinent to distributed low-power IoT environments. Meanwhile, smart contracts instituted by the blockchain guaranteed authenticated and tamper-evident actuation whenever cyber or physical disruptions are detected. Experimental validations through co-simulated hardware-in-the-loop environments confirmed the overall peak performance across fault detection (CFDA: 97.6%), control latency (avg 2.3 s), and voltage stability (± 1.1%), also ensuring resilience from peer failure and transaction flooding.

### Future scope

While the architecture as such works well in a variety of situations, several future directions remain open to exploration. Federated learning at the edge layer could serve collective anomaly detection for microgrids spread over a large geography without data coming to the center. Similarly, while the proposed blockchain architecture serves well, it can be further extended to a multi-chain or sharded architecture to improve throughput and lessen consensus delays further. Quantum-resistant cryptographic primitives may also be researched to future-proof the control layer from emerging threats. Finally, the realization of tighter observability and controllability through SDN-based adaptive routing and synchronization of digital twins then sets the stage for AI-based grid self-governance.

## Data Availability

The datasets used and/or analysed during the current study available from the corresponding author on reasonable request.
